# The early 20th century warming: Anomalies, causes, and consequences

**DOI:** 10.1002/wcc.522

**Published:** 2018-04-25

**Authors:** Gabriele C. Hegerl, Stefan Brönnimann, Andrew Schurer, Tim Cowan

**Affiliations:** ^1^ School of GeoSciences University of Edinburgh Edinburgh UK; ^2^ Oeschger Centre for Climate Change Research University of Bern Bern Switzerland; ^3^ Institute of Geography University of Bern Bern Switzerland

**Keywords:** attributing causes to climate change, decadal climate variability, extreme weather and climate events, observed climate change

## Abstract

The most pronounced warming in the historical global climate record prior to the recent warming occurred over the first half of the 20th century and is known as the Early Twentieth Century Warming (ETCW). Understanding this period and the subsequent slowdown of warming is key to disentangling the relationship between decadal variability and the response to human influences in the present and future climate. This review discusses the observed changes during the ETCW and hypotheses for the underlying causes and mechanisms. Attribution studies estimate that about a half (40–54%; *p* > .8) of the global warming from 1901 to 1950 was forced by a combination of increasing greenhouse gases and natural forcing, offset to some extent by aerosols. Natural variability also made a large contribution, particularly to regional anomalies like the Arctic warming in the 1920s and 1930s. The ETCW period also encompassed exceptional events, several of which are touched upon: Indian monsoon failures during the turn of the century, the “Dust Bowl” droughts and extreme heat waves in North America in the 1930s, the World War II period drought in Australia between 1937 and 1945; and the European droughts and heat waves of the late 1940s and early 1950s. Understanding the mechanisms involved in these events, and their links to large scale forcing is an important test for our understanding of modern climate change and for predicting impacts of future change.

This article is categorized under:

Paleoclimates and Current Trends > Modern Climate Change

## 1 INTRODUCTION

During the late 19th century, the globe was approximately 0.8 °C cooler than during the early 21st century (Hartmann et al., 2013). However, the warming between this period and the present did not proceed at a constant pace. Multidecadal phases of accelerated warming alternated with phases of slowed warming, the most recent expressions of which were the accelerated warming during the 1990s and the subsequent reduction in the global warming rate (Fyfe et al., 2016; Medhaug, Stolpe, Fischer, & Knutti, 2017; Trenberth, 2015). The ongoing discussion on the causes of that so‐called “hiatus” reveals that decadal variability in the large‐scale climate is still poorly understood, emphasizing the need for a longer‐time perspective on periods of warming and cooling during the transition to the strongly human‐influenced late 20th century climate.

One of the most prominent accelerated warming periods was the “Early Twentieth Century Warming” (ETCW) from the 1890s to the 1940s. This article discusses climate change prior to and during the ETCW, including its causes and the exceptional phenomena that were observed during this extraordinary warming period. The mid‐20th century end to the warming signaled the first period in that century that shows a detectable temperature change outside internal variability over 2–5 decades (e.g., for the trend 1916–1945 [Hegerl et al., 1996, 1997]), which then re‐emerged in trends ending in the 1990s. The ETCW was followed by a period of slow or no warming until the 1970s, which may have been partly caused by aerosol cooling, and partly by internal variability (see below; as well as Bindoff et al., 2013; Booth, Dunstone, Halloran, Andrews, & Bellouin, 2012; Jones et al., 2009; Undorf et al., 2018). Data uncertainties are larger prior to the middle of the 20th century (Morice, Kennedy, Rayner, & Jones, 2012) and are discussed as well.

The ETCW featured a pronounced Arctic warming in the 1920s and 1930s, and embedded in this period were several important climatic anomalies such as Indian monsoon failures in the 1900s (Wang, 2006; Zhou et al., 2010), the North American “Dust Bowl” droughts and record‐breaking heat waves in the 1930s (Cook, Miller, & Seager, 2009; Cowan et al., 2017; Donat et al., 2016; Schubert, Suarez, Pegion, Koster, & Bacmeister, 2004b), the cold European winters of 1940–1942 (Brönnimann et al., 2004), and the World War II period drought in Australia between 1937 and 1945 (e.g., Verdon‐Kidd & Kiem, 2009). The European summer droughts and heat waves of the mid and late 1940s (e.g., Sutton & Hodson, 2005), such as 1947 (Schär et al., 2004), followed the anomalously cold winters during WWII. These regional climatic anomalies had severe impacts on societies and the environment (e.g., Worster, 1979), and they are relevant for our understanding of the ETCW.

The ETCW received a lot of prominence from the scientific community even during the period itself (Scherhag, 1939a). Callendar (1938) attributed the warming to atmospheric CO_2_ rise. It was later discussed in the context of long‐term variability (Parker, Jones, Folland, & Bevan, 1994; Schlesinger & Ramankutty, 1994) and again in the context of the recent “hiatus” discussion. Limited observational coverage and uncertainties in forcing, particularly by aerosols (Stevens, 2015), still causes uncertainty in the attribution of the ETCW. The mechanisms involved are starting to reveal themselves as new and improved data products such as reanalyses (Compo et al., 2011) and reconstructions become available. In this article, we review the scientific understanding of the causes of the ETCW, and of regional climate trends and specific climatic events within this period. We discuss the proposed mechanisms underlying both the 50‐year warming trend and regional anomalies. Finally, we ask what we can learn from this period for our understanding of the interplay of decadal variability and increased greenhouse warming.

The first part of the article discusses the global perspective of observed climatic changes during the ETCW and its causes, followed by a discussion of regional climatic anomalies and extreme events, and conclusions.

## 2 OBSERVED LARGE‐SCALE CHANGES

Our knowledge of climate during the ETCW is mostly based on conventional weather observations at the Earth's surface, ship measurements, and some climate proxies, even though data rescue activities now aim at complementing the global climate record in this time period (Allan et al., 2011). Data coverage is further hampered by the two World Wars. Sea ice information is important, but limited in coverage. Oceanic data are equally sparse and sea‐surface temperatures (SSTs) are subject to biases, such as those the 1940s (Kennedy, Rayner, Smith, Parker, & Saunby, 2011; Thompson, Kennedy, Wallace, & Jones, 2008).

**Figure 1:**
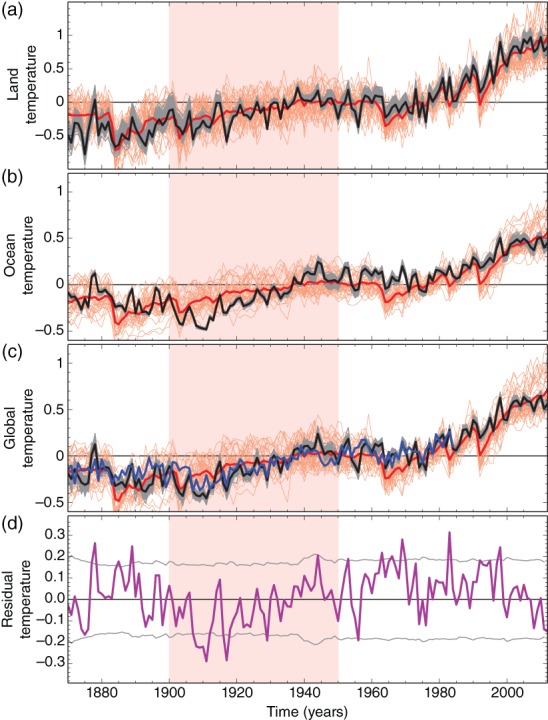
Mean surface temperature over (a) land, (b) ocean (sea surface temperatures), and (c) global (combined land + ocean) in observations (mean: black; gray lines: possible realizations given uncertainty [Kennedy et al., 2011; Morice et al., 2012]), CMIP5 multimodel mean simulations with all historical forcings (thick red line: average and thin lines: individual simulations) relative to an average over the full period. All model data are masked to observational coverage, and for combined land and sea a blend of surface air temperatures and sea surface temperature is calculated following Cowtan and Way (2014). (d) Residual variability for land and sea blend after subtracting the multimodel mean forced component, compared to 5–95% uncertainty ranges of multimodel control simulations (Method and models used the same as in Schurer et al., 2018). The blue line in panel (c) shows a global proxy‐based reconstruction (Crowley, Obrochta, & Liu, 2014) and the pink bar highlights the ETCW

Figure 1 shows globally averaged anomalies of near‐surface air temperature over land and SSTs over oceans from HadCRUT4 and a proxy‐based global scale reconstruction (Crowley et al., 2014). Other global temperature datasets show very similar changes (Hartmann et al., 2013). All show an overall warming pattern robust to data uncertainty, but with clear phases of accelerated warming from 1900 to the 1940s and from the 1970s to 2000s, a stagnation from the 1940s to the 1970s, and a slowdown in warming from 2000 to about 2013. A period of anomalously cold conditions at the beginning of the ETCW period is particularly pronounced in sea surface temperatures (Figure 1b). Estimates of the uncertainty in a single dataset are visible but not large (Morice et al., 2012; see Figure 1), and simulations that contribute to the Coupled Model Intercomparison Project Phase 5 (CMIP5)’s multi‐model ensemble reproduce many of the key observed features. Figure 1 compares climate models and observational data over the regions where observations are available, and uses a combination of SSTs over ocean and near‐surface air temperature over land in both models and data in order to reduce biases in model‐data comparisons (Cowtan & Way, 2014). A truly global average of temperature would in addition be affected by missing data, particularly in the Arctic (Karl et al., 2015). Even fairly recently, an inhomogeneity of SST data, caused by changing contributions by different shipping fleets, has been detected and corrected (Kennedy et al., 2011), while other inhomogeneities are likely still present, which may lead to an overestimate of the unforced component of the ETCW (Chan & Huybers, n.d.). In addition, there are remaining biases in land temperature data, such as summer temperature biases preceding the widespread use of the Stevenson screen (Auchmann & Brönnimann, 2012; Brunet et al., 2011) which may lead to an overestimation of summer temperatures prior to the second half of the 20th century. Processing techniques such as gridding methods are less important, since using different statistical approaches lead to very similar global mean temperatures (Rohde et al., 2013).

Support for the large‐scale temperature record during the past 150 years comes from recently constructed, noninstrumental global temperature index based on proxies (Anderson et al., 2013; Crowley et al., 2014), and other proxy reconstructions of Northern Hemispheric temperatures that also show the early 20th century as a pronounced period of warming (see Figure 1c). Thus, although there may be remaining uncertainties on the exact temperature the ETCW started from, and peak temperatures during it, the early 20th century showed robust global warming.

**Figure 2:**
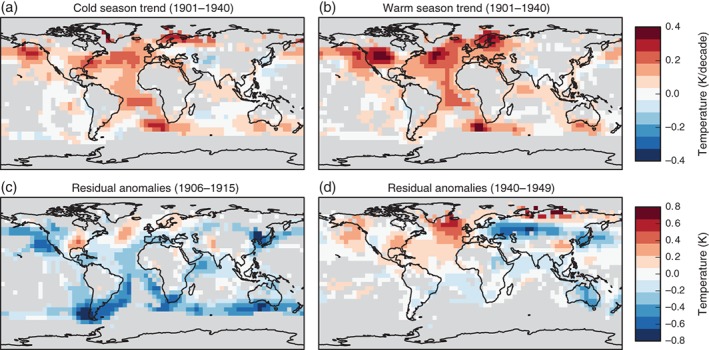
Spatial maps showing decadal temperature trends for boreal cold (a, November–March) and warm season (b, April–October) for the early 20th century (1901–1940) in HadCRUT4 (Morice et al., 2012). (c, d) Spatial temperature anomalies from HadCRUT4 after the multimodel mean CMIP5 response to all historical forcings combined has been subtracted based on analysis discussed below. All panels are masked where coverage is too sparse (gray); in panels (a) and (b) where coverage in either the first or second half of the period drops below 30%; in panels (c) and (d) where coverage in any season drops below 30% in either the first or second half of the decade. For details see Supporting information

The spatial pattern of warming (Figure 2) is less well resolved in many regions of the world than the recent warming, with many key regions missing or only covered by sporadic measurements, for example, the tropical Pacific and much of the Southern Hemisphere, as well as the African interior, South America, and Asia (Figure 2). Nevertheless, many robust features emerge: in the HadCRUT4 annual and seasonal mean surface air temperature, the warming over the first four decades of the 20th century appears particularly prominent over high latitudes of Europe, the Atlantic and over the northern North Pacific and Canada (the warming from 1901 to 1951, used in the attribution analysis, is similar in pattern although with slightly less pronounced anomalies, Figure S1; Figure S2 for model fingerprints). Unusual early warmth in the Arctic occurred as early as 1918, discussed further below. There is also a remarkably strong temperature trend over the Atlantic, peaking in the midlatitudes, near the equator and the far North, while parts of Eurasia are neutral or cooling. This trend is particularly pronounced in boreal summer. The ETCW pattern is distinctly different from the widespread warming pattern over the entire 20th century (Hartmann et al., 2013) in both the boreal cold and warm seasons. The latter strongly resembles the expected fingerprint of greenhouse warming with stronger warming over land than oceans, and enhancement in the Arctic (Bindoff et al., 2013; Brönnimann, 2009).

Precipitation changes over the early 20th century are highly uncertain with only a limited amount of in situ data available over land (see Hegerl et al., 2015, and references therein) including some island stations that extend back into the early 20th century (Polson, Hegerl, & Solomon, 2016). Figure S3 shows an estimate of precipitation trends for the 1901–1945 period for the boreal warm (April–September) and cool (October–March) precipitation seasons (Schneider et al., 2014). Despite high data uncertainty, some features are consistent with changes in precipitation that are expected with warming, namely increased precipitation in the region of the Intertropical Convergence Zone (ITCZ) and in northern high latitudes but decreased precipitation in the outer tropics and parts of the subtropics.

**Figure 3:**
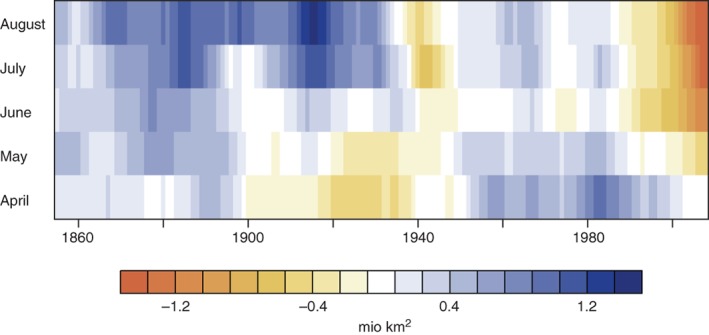
Anomalies in Arctic Sea ice extent (million km^2^) from Walsh et al. (2017) in the months of April through to August relative to the climatology from 1931 to 1960, smoothed with a 10‐year moving average

The ETCW was both a period of Arctic warming and Arctic sea ice retreat. This retreat is difficult to document, as early observational data are often infilled with climatology in regions without measurements, leading to a possible underestimation of sea ice retreat during this period. Recent reprocessing adding more ice chart data has made the early 20th century ice retreat more visible and well documented at least in some regions (Titchner, personal communication, 2017; Walsh, Fetterer, Stewart, & Chapman, 2017), but the limited availability of observations is still problematic (see Fig. 5 in Walsh et al., 2017). Figure 3 illustrates a substantial reduction of the best‐sampled spring and early summer ice around 1940. Climate models tend to show much weaker sea ice reductions over the ETCW period (H. Titchner and D. Polson, personal communication, September 11, 2017), suggesting that, similar to the Arctic atmospheric warming, the observed retreat is driven at least in part by internal climate variability (Brönnimann et al., 2012; Wood & Overland, 2010). This early sea ice retreat period is an important yardstick for the recent ice retreat and ice‐temperature relationships. Hence further processing of old charts and reducing the infilled parts of the sea ice record is vital.

## 3 CAUSES AND MECHANISMS OF THE EARLY 20TH CENTURY WARMING

**Figure 4:**
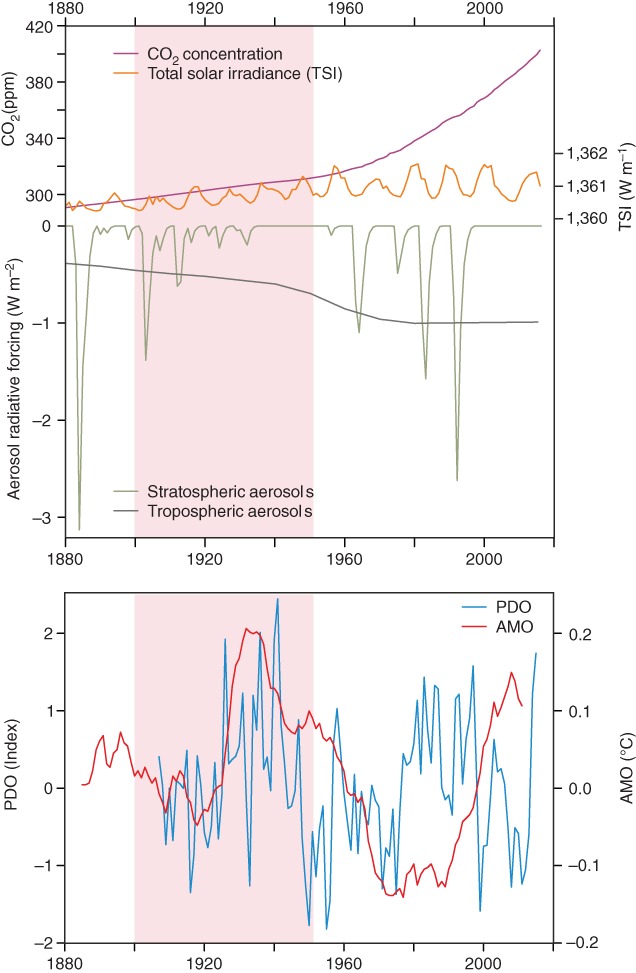
(Top) Annual mean time series of climate forcings agents: CO_2_ concentrations (from reconstructions [Yoshimori, Stocker, Raible, & Renold, 2005] and Mauna Loa measurements, NOAA), total solar irradiance (TSI; Coddington, Lean, Pilewskie, Snow, & Lindholm, 2016); note that effective solar forcing of 1 W/m^2^ in TSI translates to ca. 0.175 W/m^2^ when correcting for insulated area and albedo, which is accounted for by using a different scale), as well as estimated forcing from stratospheric and tropospheric aerosols (from NASA/GISS CMIP5). (Bottom) Indices of the Pacific Decadal Oscillation (PDO) and the Atlantic Multidecadal Oscillation (AMO). The AMO index is defined (Trenberth & Shea, 2006) as the difference in SST averages over the regions (0–60°N, 0–80°W) and (60°S–60°N). The PDO is calculated by projecting the first Empirical Orthogonal Function mode onto the median realization of HadSST3.1.1.0 at 5° resolution (from the Climate Explorer, following Mantua, Hare, Zhang, Wallace, and Francis (1997)

What caused the long‐term warming from 1900 to 1950? The global temperature rise during the ETCW implies a change in the energy budget of the Earth's atmosphere, which in turn suggests either an external forcing (volcanic, solar, greenhouse gases, tropospheric aerosols), changes in clouds, or ocean heat release (Brönnimann, 2015b). Figure 4 shows that changes in several external forcings over the ETCW could be important, such as: a greenhouse gas increase, a small change in solar irradiance, and a reduction in stratospheric aerosols associated with reduced volcanic activity.

Solar irradiance slightly increased from about 1900 onward (the period around 1890 is sometimes called the “Gleissberg minimum”), although the magnitude of this increase is uncertain (Coddington et al., 2016) with an overall forcing estimate shown in Figure 4 of about 0.25 W/m^2^, which is on the large side compared to other estimates (Wang, Lean, & Sheeley Jr, 2005). The magnitude and pattern of the response to an increase in solar forcing is highly uncertain (Gray et al., 2010). A detection and attribution analysis of temperature reconstructions of the past centuries suggests a rather small influence of solar variability on hemispheric scales (Schurer, Tett, & Hegerl, 2014), which, however, does not preclude regional and seasonal effects such as an influence on circulation contributing to cold European winters (Lockwood, Harrison, Woollings, & Solanki, 2010).

Tropical volcanic eruptions, which were frequent in the 19th century (e.g., Tambora in 1815, and Krakatoa in 1883), became much rarer after the Santa Maria eruption of 1902 and the 1912 Katmai eruption. The relative muting of eruptions between this period and the eruption of Mount Agung in 1963/1964 thus implies a positive forcing, although volcanic emissions during this period are not well known (Neely III & Schmidt, 2016). Epoch analyses suggest that periods without volcanic forcing show warming as the climate relaxes back into a less volcanically cooled background state (Hegerl, Crowley, Baum, Kim, & Hyde, 2003; Schurer, Hegerl, Mann, Tett, & Phipps, 2013), and climate models show long‐term climate change in response to changes in the statistics of eruptions (Gregory, 2010; Schurer et al., 2014).

The CO_2_ concentration increased from the beginning of industrialization in the 18th century, continuing to increase over the early 20th century and then accelerating more recently. Since CO_2_ forcing is logarithmic with concentration, early forcing is disproportionally important. Callendar (1938) already attributed the ETCW to increased CO_2_ concentration in the late 1930s. Some of the effect of CO_2_ increases should have been counteracted by an increase in anthropogenic aerosols, which were important already over this period (Undorf, Bollasina, & Hegerl, 2018; Undorf, Polson, et al., 2018), and alone should have caused a substantial cooling effect. However, even by 1900, reconstructions of hemispheric temperatures show evidence for a detectible warming driven by increases in greenhouse gases, particularly relative to slightly reduced CO_2_ during the Little Ice Age (Abram et al., 2016; Schurer et al., 2013) consistent with attribution of a substantial fraction of the ETCW in temperature reconstructions to greenhouse gas increases (Schurer et al., 2013). This is in addition to the warming over much of the 19th century that was driven by the recovery from an active volcanic period early in that century (Brönnimann & Krämer, 2016; Raible et al., 2016).

A further possible contributor to the ETCW is decadal variability in the climate system. Figure 2 shows that the ETCW involves pronounced SST anomalies; with anomalously warm Atlantic temperatures both in the boreal cool and warm season; and more neutral land temperatures on average (where covered). The pronounced Atlantic warming can be interpreted in terms of shifts in multidecadal oceanic or coupled variability modes (see Hartmann et al., 2013), most notably the Atlantic Multidecadal Oscillation (AMO; Schlesinger & Ramankutty, 1994). The AMO is usually described as the smoothed basin‐averaged SST anomalies in the North Atlantic, although its physical mechanism is not fully clear. It has been suggested that the AMO expresses the strength of the meridional overturning circulation of the Atlantic Ocean (Knight, Allan, Folland, Vellinga, & Mann, 2005; McCarthy et al., 2015). Model studies on the other hand suggest that many features of the AMO can be reproduced without a dynamic ocean (Clement et al., 2015; Srivastava & DelSole, 2017). While some authors emphasize an effect of AMO‐related SST anomalies on the atmosphere (Gastineau & Frankignoul, 2015), many recent studies emphasize the atmosphere as a driver (Häkkinen, Rhines, & Worthen, 2011), or portray a “delayed‐oscillator”‐type coupled mode (Sun, Li, & Jin, 2015). With respect to forcings, the AMO appears to be partly driven by external forcing (Faurschou Knudsen, Jacobsen, Seidenkrantz, & Olsen, 2014), including possibly aerosol forcing (Booth et al. (2012), although this has been questioned (Zhang et al., 2013), as well as to volcanic (Otterå, Bentsen, Drange, & Suo, 2010) and solar forcing (Malik, Brönnimann, & Perona, 2017). This complicates attribution of observed change to forcing or internal variability.

There is also evidence for a role of the Pacific Decadal Oscillation (PDO, Mantua et al., 1997) during some of the ETCW (Tokinaga, Xie, & Mukougawa, 2017), although data coverage is not great and uncertainties are substantial (see Fig 2 in Tokinaga et al. (2017). The PDO is here defined as the leading mode of North Pacific SST anomalies North of 20°N (Mantua et al., 1997), and is related to the decadal realization of the El Niño‐Southern Oscillation (ENSO; Zhang, Wallace, & Battisti, 1997; Cai, van Rensch, Cowan, & Sullivan, 2010) and probably describes a combination of processes, some of which operate on different time scales (Newman et al., 2016).

The PDO and AMO indices both increased during the ETCW (Figure 4, bottom panel), and remained elevated, peaking in the early 1940s. The AMO index increased sharply in the 1920s, peaked around 1930 yet remained high in the 1940s. Thus, the period of the ETCW coincides with upward trends in both modes. From observations alone it is difficult to determine the role of these decadal modes on climatic anomalies during the ETCW due to the short record, although there is evidence that both PDO and AMO contribute to phenomena observed during the ETCW, such as the US drought in the 1930s (McCabe, Palecki, & Betancourt, 2004).

## 4 ATTRIBUTION OF EARLY 20TH CENTURY WARMING

Detection and attribution studies are used to disentangle the effect of external forcings on observed climate by quantifying the fingerprint of responses to each of the forcings relative to each other and internal variability (Bindoff et al., 2013), including multidecadal variability (which is, at least to some extent, simulated in climate models [Flato et al., 2013]). Attribution studies analyzing the entire instrumental record have indicated that some of the large‐scale observed warming in the early 20th century exceeds what is expected from internal climate variability and was forced (Bindoff et al., 2013; Gillett, Arora, Matthews, & Allen, 2013; Hegerl et al., 1996, 1997; Ribes & Terray, 2013). However, different analyses emphasize different contributors to the warming (Bindoff et al., 2013). For example, Shiogama, Nagashima, Yokohata, Crooks, and Nozawa (2006) found a contribution from solar and volcanic forcing in the global temperature record, while Hegerl et al. (2007) attributed the ETCW in Northern Hemisphere temperature reconstructions to greenhouse gas increases and decrease in volcanic forcing combined with internal climate variability. Others studies emphasize the strong role of internal variability (Delworth & Knutson, 2000; Ring, Lindner, Cross, & Schlesinger, 2012), consistent with the observed 1918–1940 warming being significantly greater than that simulated by most of the CMIP3 models (Crook & Forster, 2011).

**Figure 5:**
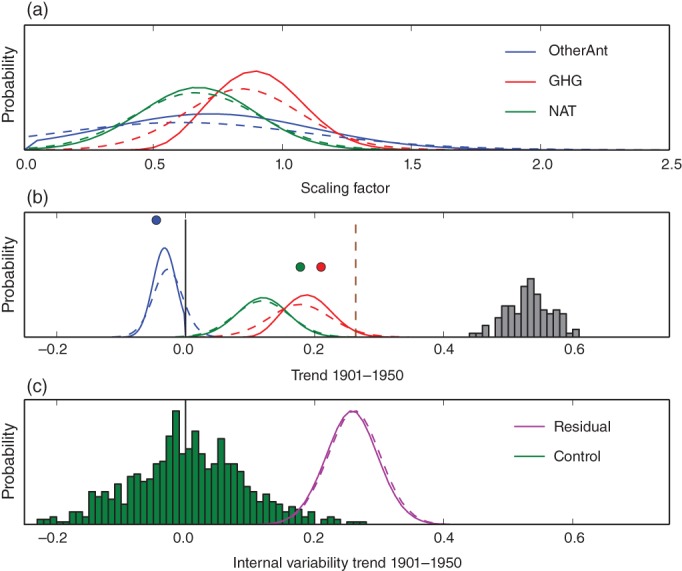
Top panel: Scaling factors, that is, magnitude of model fingerprint consistent with observations for the response to greenhouse gases (red), other anthropogenic factors (blue) and natural factors (solar and volcanic, green) from an analysis of decadal temperatures over 1862–2012. This translates into an estimated contribution to warming 1901–1950 by increases in greenhouse gases, natural forcings, and other anthropogenic forcings compared to the ensemble of HadCRUT observed warming accounting for uncertainty (gray histogram) shown in the middle panel. Dots: Multimodel unscaled best estimate of contribution by forcing. pdfs: Estimated contribution from attribution analysis allowing for up‐ or down‐scaling as long as consistent with observations. Dashed line: Trend from a proxy reconstruction (Crowley et al., 2014). Bottom panel: Residual warming not explained by forcing (purple pdf) compared to CMIP5 control run trends over 50 years to estimate internal variability (green). All analysis is done with model data masked to replicate the data coverage of observed data, and merging sea surface temperature with air temperature over land (Cowtan et al., 2015), solid lines show results using an informative prior, dashed lines an uninformative prior. After Schurer et al., 2018

Figure 5 illustrates contributions to ETCW based on a recent detection and attribution analysis for the instrumental record (1863–2012). The results suggest that all factors discussed above contributed: anthropogenic forcing, natural forcing, and anomalous climate variability.

The analysis is conducted by finding a combination of model derived time–space patterns of response to greenhouse gas, other anthropogenic and natural forcing (so‐called fingerprints) that best matches the observations over the entire instrumental period (see Supporting information for more detail). The amplitude of each fingerprint that is consistent with data is estimated as a distribution of “scaling factors” (top panel in Figure 5), which when multiplied by the raw model results yields a distribution of the contribution of each forcing to the total observed anomaly (middle panel) and a residual variability not explained by forcings (bottom panel; see also Figure 1, bottom panel and Hegerl & Zwiers, 2011 for more detail). The analysis indicates that both external forcing and internal climate variability contributed to the observed warming, with a strong greenhouse warming contributing substantially to the ETCW, natural forcings contributing as well, and other anthropogenic forcing agents such as aerosols counteracting the warming. The natural signal is, at least in climate model simulations, largely due to a hiatus in volcanic eruptions (see Figure S4). Overall, about a half (40–54%; *p* > .8) of the global warming from 1901 to 1950 is estimated to have been forced. Forcings can, however, not fully explain the warming (indicated by the histogram of observed, positive, residual trends, Figure 5; compare also Figure 2 top panels with Figure S2). Note that the residual from a proxy‐based global reconstruction is much smaller (Crowley et al., 2014), raising questions if the strong bias of observed data toward the Atlantic sector enhances the residual warming. This residual warming is, however, not extraordinary relative to samples of 50‐year trends in climate models. Figure 5 also shows that there is substantial data uncertainty in the strength of the warming over the data covered part of the globe, indicated in the gray histogram of observed trends. Note that the analysis is conducted utilizing only data covered parts of the globe; and that model SST data are used over ocean and combined with surface air temperature (Cowtan & Way, 2014) for best comparability with HadCRUT data (see Appendix S1 for more details of the analysis).

## 5 ROLE OF CLIMATE VARIABILITY AND SEA SURFACE TEMPERATURES

**Figure 6:**
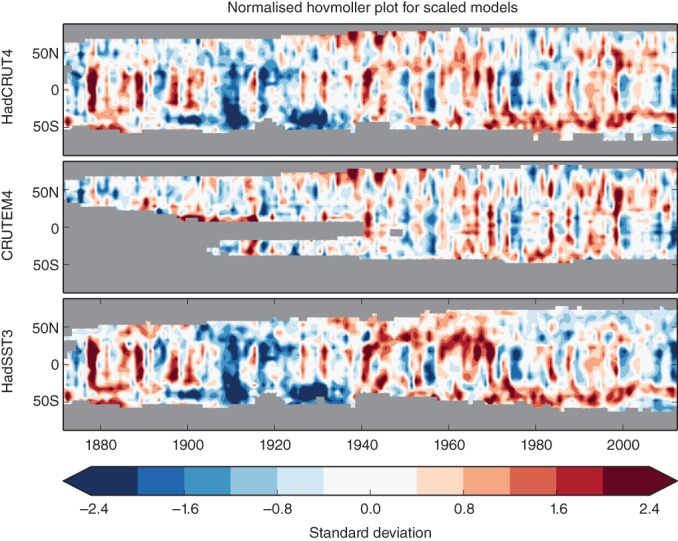
Top panel: Hovmoeller diagram of standardized zonal mean temperature anomalies over land and ocean (top), land only (middle) and sea surface temperature (bottom) after the multimodel mean forced signal, represented by the average of model simulations forced by historical forcing has been subtracted. The diagram is in standard deviation units of zonal mean variability in the data‐covered parts of each zone relative to multimodel interannual variability from control simulations. Data are masked when data coverage for a particular zonal band drops below 10% (shown in gray; see Figure S6 for a version restricted to >30% coverage)

Given external forcing can only partly explain the ETCW (see above, see also Ting, Kushnir, & Li, 2014), we now address which modes of variability may have contributed. When removing the multimodel mean of the response to all historical forcings combined (or similar, when removing optimal fitted fingerprints, not shown), a substantial residual variability remains (Figures 1d and 5c). This variability is investigated more closely in Figure 6, showing that there is substantial involvement of both the tropics and extratropics in warming and cooling periods related to decadal climate variability. It also highlights that the ETCW started from a very unusual cold anomaly in the first decade of the 20th century, originating both from tropical and Southern Hemisphere SSTs (Figure 6). These cool anomalies began in the South Atlantic, with some land datapoints also showing cool conditions, and then spread globally in the subsequent decade (Figure 2, bottom) leading to cold anomalies in both Atlantic and Pacific (see also Figure S5). This rarely discussed cold period was followed by strong warming in the Northern Hemisphere, which was particularly pronounced in high latitudes.

What mechanisms might be linked to the strong residual warming? Using proxy data, Thompson, Cole, Shen, Tudhope, and Meehl (2015), suggested that a PDO warm phase served as a trigger for the ETCW through weaker Pacific Trade winds. This is supported by targeted model simulations (Tokinaga, Xie, Deser, Kosaka, & Okumura, 2012) and is consistent with several recent studies suggesting that tropical Pacific SSTs play a key role as pacemakers for decadal periods of fast and slow warming (England et al., 2014; Meehl, Arblaster, Fasullo, Hu, & Trenberth, 2011; Roberts, Palmer, McNeall, & Collins, 2015; Schurer, Hegerl, & Obrochta, 2015). Ocean interbasin energy exchange makes it difficult to attempt to trace the location of heat uptake (Chen & Tung, 2014; Drijfhout et al., 2014; Hedemann, Mauritsen, Jungclaus, & Marotzke, 2017; Lee et al., 2015). Gaps in data coverage cause higher uncertainty during the early 20th century than for recent periods. Particularly, very little information is available on the Indo‐Pacific and Southern Oceans during that period, and none for the ocean interior.

Several questions arise: to what extent was the Atlantic Ocean driving an anomalous atmospheric circulation contributing to Arctic warming, and to what extent was the atmosphere driving the ocean. While many publications consider the AMO as an internal oceanic mode (Chylek, Folland, Lesins, Dubey, & Wang, 2009; Knight, 2009; Tung & Zhou, 2013), others argue that the SST anomalies in the Atlantic Ocean during the ETCW were largely driven by anomalous atmospheric circulation (Wood & Overland, 2010), consistent with the idea that atmospheric variability drives decadal ocean‐related variability (Hasselmann, 1976). Forcing an ocean model with wind fields from the 20th century reanalysis (20CR), Müller et al. (2015) reproduced important features (e.g., salinity anomalies) of the North Atlantic Ocean anomalies in the early 20th century, and others find a contribution from the Pacific through a shift in the Aleutian low (Overland & Wang, 2005) to the Arctic warming and a contribution of the Pacific sector to poleward heat advection into the Arctic particularly during the early phase (1910s and 1920s) of the Arctic warming (Wegmann, Brönnimann, & Compo, 2017).

Overall, SSTs over both the Atlantic and Pacific sectors are required to reproduce the Arctic warming in recent simulations (Ting et al., 2014; Tokinaga et al., 2017). Independent of why the Arctic warmed, most studies also agree that the warming did affect atmospheric circulation by shifting the ITCZ and the entire northern Hadley circulation poleward (Brönnimann, 2015a; Ting et al., 2014).

## 6 CHANGES IN ATMOSPHERIC CIRCULATION

**Figure 7:**
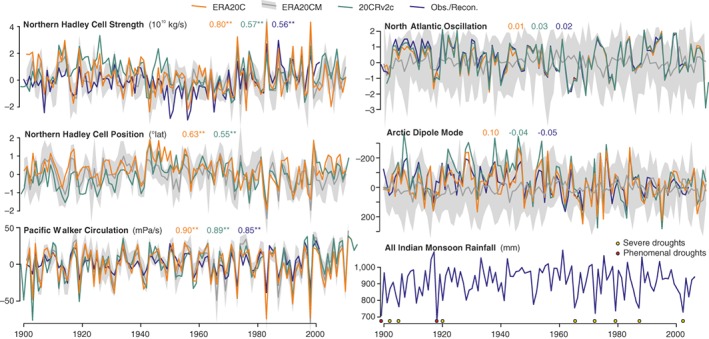
Annual time series of circulation indices, expressed as anomalies from 1961 to 1990. Shown are series from two reanalyses (20CRv2c, ERA20C), the atmospheric model simulations ERA20CM (10 members; ensemble mean and spread are indicated (Hersbach et al., 2015) and series based on observations or reconstructions. Shown are the seasonal mean strength of the boreal Hadley cell (the maximum of the meridional mass stream function), its latitudinal position (latitude of the maximum), the Pacific Walker cell (difference in 500 hPa *ω* between the areas [10°S–10°N, 180–100°W] and [10°S–10°N, 100–150°E]), the North Atlantic oscillation (NAO) and the Arctic dipole mode (here defined as the SLP difference north of 60°N between the western and the eastern hemisphere; after Brönnimann et al. (2009) and Brönnimann et al. (2009). Additionally, a statistical reconstruction of the Pacific Walker cell is shown (Brönnimann, Stickler, Griesser, Ewen, et al., 2009; Brönnimann, Stickler, Griesser, Fischer, et al., 2009) and an observation‐based NAO index based on HadSLP2p data (Allan & Ansell, 2006). All series are for December–February except Hadley cell position (January–December) and Indian summer monsoon rainfall (June–August, Sontakke et al., 2008). Colored numbers indicate correlations between the correspondingly colored reanalyses or observations with the ERA20CM ensemble mean (* and ** denote 95 and 99% significance). The purple line for Indian summer monsoon rainfall is based on a reconstruction (Zhou et al., 2010). Severe and phenomenal droughts (Wang, 2006) over India are added as circles

Short‐term climate variability changes the probability of extreme events (Kenyon & Hegerl, 2008; National Academies of Sciences, Engineering, and Medicine, 2016) and can both show decadal preferences itself and cause longer‐term changes in slower climate components (Hasselmann, 1976). Recent reanalysis efforts such as 20CR (Compo et al., 2011) provide researchers with the means to address the causes of atmospheric circulation changes over the 20th century and the role of SSTs in driving such changes. Figure 7 shows the seasonal mean strength of key circulation indices such as the boreal Hadley cell, its latitudinal position, the Pacific Walker cell, the North Atlantic Oscillation (NAO) and the Arctic Dipole Mode from observations, reconstructions, and two historical reanalyses (20CR version 2c [Compo et al., 2011], and ERA‐20C [Poli et al., 2016]) and from an ensemble of 10 atmospheric climate model simulations from ERA‐20CM (Hersbach et al., 2015). When the SST driven simulations reproduce the index this indicates a strong driving role for SSTs.

The boreal winter Hadley cell appears to have weakened from the 1920s into the 1950s, then strengthened until the late 1990s. However, uncertainties even in the latter signal are large (Hartmann et al., 2013). The position of the northern tropical belt shifts poleward until the early 1940s, then equatorward until the 1980s (Brönnimann, 2015a), followed again by a slight poleward shift. For both indices, different data sets agree, and they agree with atmospheric general circulation model (GCM) simulations (Brönnimann, 2015a), which implies that these changes are consistent with and influenced by SSTs. In fact, from the stronger warming of the Northern Hemisphere relative to the Southern Hemisphere, we expect a poleward shift of the northern Hadley cell.

Dependence on SSTs is also found for the Pacific Walker circulation, which is closely related to ENSO, with interannual variability being more prominent than decadal variability. Individual events such as the 1918/1919 El Niño event (Giese et al., 2010) or the 1939–1942 El Niño appear prominently in all series, and there is proxy evidence for a decrease of the trade winds over the central equatorial Pacific (i.e., a weakening of the Walker circulation) in the 1910s (Thompson et al., 2015). This is not inconsistent with the indices displayed, although it is not a prominent feature in Figure 7. Less frequent El Niño conditions (indicated by a high Walker Circulation index) are found around 1910, in the early 1920s, the 1930s, the 1950s, the 1970s, and the 2000s; these coincide with negative PDO phases (Figure 4, bottom panel).

The Indian Monsoon, according to a reconstructed Dynamic Indian Monsoon Index was rather strong in the 1880s (Figure S7, see Zhou et al., 2010), then dropped into a pronounced minimum from around 1900 to 1915 near the start of the ETCW. This coincides with minimum Indian Monsoon summer rainfall and occurrence of droughts (Figure 7).

The NAO index was high until the mid‐1920s (Rogers, 1985), then decreased to the 1960s, followed by a strengthening to the mid‐1990s (Hurrell, 1995). Hence, during the latter part of the ETCW, the winter circulation over the North Atlantic weakened. This suppressed some of the winter warming that would have occurred otherwise over this time period, and likely also influenced SSTs (Iles & Hegerl, 2017), hence the NAO downward trend not only does not contribute to the early 20th century warming but slightly counteracted it. Different NAO indices agree well, but are not well reproduced by the SST driven GCM (Scaife et al., 2009), consistent with a largely atmospheric origin of low‐frequency NAO variability.

Decadal changes are also found in the Arctic Dipole Mode (here shown for the annual mean). The index decreased (note the inverted scale in Figure 7) from the late 1910s to the 1940s. During the latter part of the ETCW (Grant, Brönnimann, Ewen, Griesser, & Stickler, 2009; Overland & Wang, 2005; Wegmann et al., 2017), positive pressure anomalies over Eurasia contrasted with negative anomalies over North America. This is consistent with a tendency toward warm air advection into the European Arctic and hence Arctic warming. In the 1930s, circulation related to a negative Pacific‐North American (PNA) pattern also allowed poleward transport of warm air masses over the northwestern Pacific (Wegmann et al., 2017), (see also Figure S7). A strong positive peak in the PNA then followed in the early 1940s (Ewen, Brönnimann, & Annis, 2008), which brought warm air toward western Canada and into Alaska, followed by an abrupt drop in the 1950s. Both these circulation anomalies contributed to Arctic warming.

In conclusion, pronounced interannual and, to some extent, decadal atmospheric variability occurred over the ETCW period, including a poleward shift of the northern Hadley cell, and a weakening of the NAO and of the Arctic Dipole Mode. While these circulation anomalies are linked to regional anomalies during portions of the ETCW, none of them provides a strong contribution to the large‐scale warming over the entire ETCW.

## 7 EXTREME WEATHER AND CLIMATE EVENTS

The ETCW encompasses several prominent climatic anomalies, which have mostly been studied in isolation, but form an integral part of the ETCW. For example, warm North American summer anomalies and Arctic winter anomalies make a large contribution to the early 20th century scale average anomaly (Brönnimann, Stickler, Griesser, Ewen, et al., 2009; Brönnimann, Stickler, Griesser, Fischer, et al., 2009). While extreme events are often associated with unique weather systems or local forcings, large‐scale factors such as those discussed above can substantially influence the probability of unusual events (National Academies of Sciences, Engineering, and Medicine, 2016). For example, greenhouse gas increases have significantly increased the probability of some recent heat wave events (Stott et al., 2016). It is important to understand the mechanisms and forcing involved in events prior to the middle of the 20th century, since early events provide a test for our understanding of climate change, evaluate the range of extreme events simulated by present climate models, and provide test cases for impacts associated with extreme events. Yet analysis of these early events is still in its infancy. In the following section this review article describes six major climatic anomalies in chronological order, and relates them to large‐scale factors where possible, summarizing in a schematic figure at the end of the article.

### 7.1 The weak Indian monsoon 1890s–1910s

The Indian Summer Monsoon rainfall was below normal in the 1890s–1910s (Goswami, Madhusoodanan, Neema, & Sengupta, 2006; see Figure 7) consistent with low values in the Dynamic Indian Monsoon index (Figure S7). A complete monsoon failure (phenomenal drought according to Wang (2006), accompanied by a devastating famine) occurred in 1899. Severe droughts affected India also in 1902, and 1905. As a direct result of these events John Eliot, director of the India Meteorological Department, hired Gilbert Walker to analyze (and possibly forecast) monsoon variability. This period of weak monsoons ended after another complete monsoon failure (phenomenal drought) in 1918 and a severe drought in 1920.

These anomalous monsoons are linked to large scale anomalies discussed above. The Indian Summer Monsoon has been shown to be affected by SSTs in the Atlantic (Goswami, Madhusoodanan, Neema, & Sengupta, 2006; Krishnamurthy & Krishnamurthy, 2016) and the Pacific. Specifically, decadal phases of monsoon failure in climate model simulations have been found to coincide with phases of negative AMO and concurrent positive PDO (Malik et al., 2017). The AMO may be linked to the Indian monsoon through changes in the summer NAO and Eurasian temperatures, which affect the meridional temperature gradient over south Asia (Goswami, Madhusoodanan, Neema, & Sengupta, 2006). The downwelling branch of the Indian Ocean Walker circulation over the Indian subcontinent is strongest when uplift in the tropical west Pacific is strongest, linking to the Walker circulation through the developing phase of ENSO events over the summer. El Niño events with largest SST anomalies near the dateline are more likely to produce a weak Indian monsoon (Krishna Kumar, Rajagopalan, Hoerling, Bates, & Cane, 2006), although there is considerable variability in the ENSO‐monsoon relationship and the monsoon system itself (Sinha et al., 2015). SST anomalies in the Indian Ocean during the 1899–1918 period correspond to a positive mode of the subtropical Indian Ocean dipole (Saji, Goswami, Vinayachandran, & Yamagata, 1999). The monsoon failure around 1900 coincides with a negative phase of the AMO (Figure 4), which is consistent with the mechanisms outlined above. The PDO phase is more uncertain during this period, although there is evidence to suggest that it may be have been in a weak positive phase (McCabe, Palecki, & Betancourt, 2004). Hence, the weak Indian monsoon in the 1900s perhaps partly reflects the cold Atlantic–Eurasian sector, which is the starting point from which the ETCW then unfolded.

### 7.2 The warming of the Arctic from the 1910s to the 1940s

After 1918 a pronounced warming started in the northern North Atlantic region (e.g., Yamanouchi, 2011). In Spitsbergen, the average temperature of the years 1919–1924 was 3 °C higher than in the years 1913–1918 (Grant et al., 2009). The step‐like temperature increase was noted by contemporary scientists, but no obvious change could be found in the instrumentation or station surroundings, and the change concurred with a decrease of sea ice (Birkeland, 1930; Scherhag, 1939a, 1939b). A recent homogenization (Nordli, Przybylak, Ogilvie, & Isaksen, 2014) also did not remove the step change. Unlike the recent Arctic warming period, which is strongest near the ground (Grant, Brönnimann, & Haimberger, 2008), the Arctic warming in the European sector in the 1920s was most pronounced in the rather sparse upper‐air data at 700 hPa (Brönnimann et al., 2012; Grant et al., 2009). Temperatures then further increased until the late 1940s, with an interruption in the 1940–1942 period. A rapid transition toward lower temperatures occurred in the late 1940s, and is particularly strong in reanalysis data at 700 hPa in winter (Wegmann et al., 2017).

**Figure 8:**
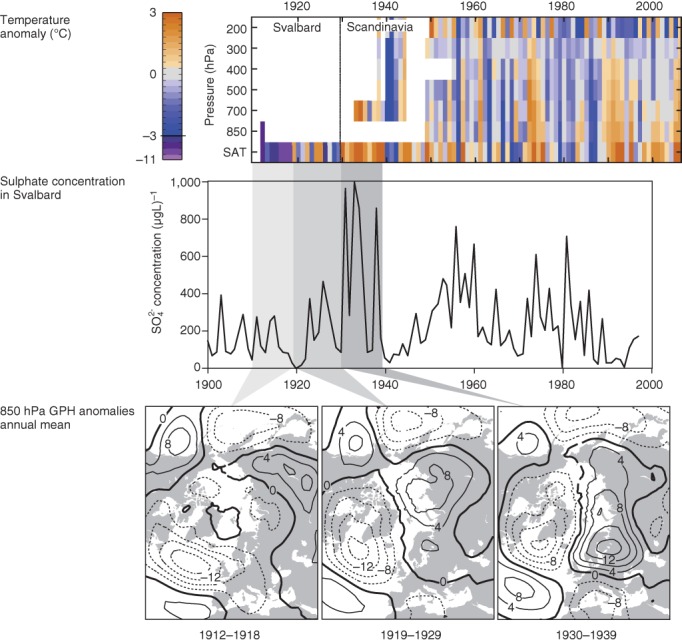
Top panel: Time‐height cross section of seasonal‐mean temperature anomalies as a function of pressure and year for the European Arctic in winter. Middle panel: Sulfate concentrations as a function of time from the Lomonosovfonna ice core (Svalbard). Bottom panel: Reconstructed 850 hPa geopotential height anomalies (relative to 1961–1990) for annual means in (left) 1912–1918, (middle) 1919–1929, and (right) 1930–1939 (from Grant et al., 2009)

The step change in temperature in 1918/1919 coincides with a tendency toward a pressure dipole over the Atlantic Sector that leads to southerly flow into the Arctic (Figure 8, which is sometimes termed a negative trend in the Barents Oscillation and related to the Arctic dipole discussed above, Figure 7). This is not only found in reanalysis data sets and reconstructions, but is also reflected in (trace) aerosols in an ice core from Svalbard (Grant et al., 2009). Spikes in the sulfate concentration in the 1930s point to increased transport of polluted air from central and Western Europe toward the Arctic, in very good agreement with circulation reconstructions, and also increased transport of black carbon aerosols, presumably from North America, to Greenland during the 1910s–1930s (McConnell et al., 2007).

As discussed in the previous section, the anomalous warming in the European Arctic was arguably related to the warm North Atlantic (see also Fyfe et al., 2013), and extratropical North Pacific (Tokinaga et al., 2017), yet the exact role of the atmosphere versus the ocean is unclear. Simulations with coupled climate models support that a large fraction of the Arctic warming is caused by internal climate variability and can occur in large climate model ensembles (Beitsch, Jungclaus, & Zanchettin, 2014). However, a small part of the Arctic winter warming is also linked to external forcing, particularly anthropogenic forcing (see Figure S2), consistent with Fyfe et al. (2013) and attribution results. Feedback mechanisms, such as feedbacks caused by sea‐ice retreat (see Figure 3) or aerosols on snow (see Figure 8) may have strengthened the extraordinary warming in the Arctic during the 1920s and 1930s.

### 7.3 The Dust Bowl droughts and heat waves of the 1930s

**Figure 9:**
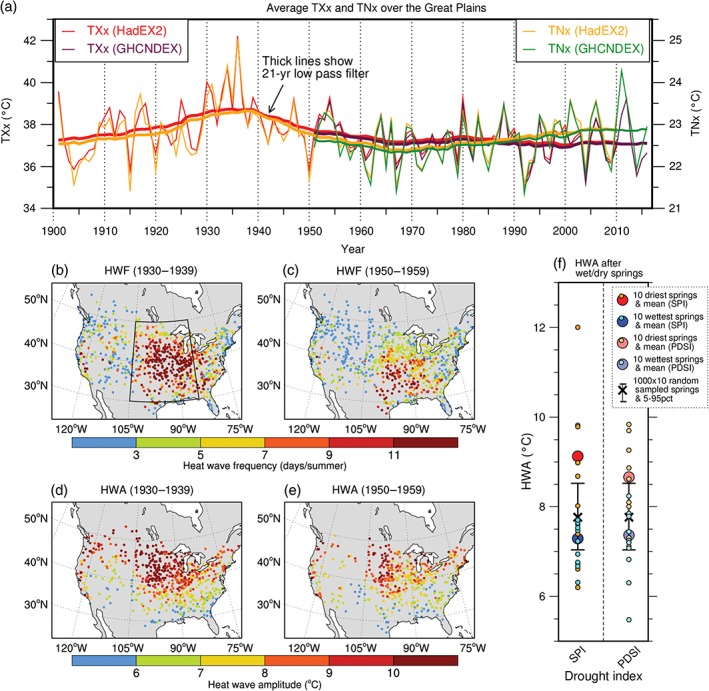
Record heat waves in the Midwestern United States and the role of spring drought: (a) time evolution of maximum of daily maximum (TXx) and minimum (TNx) temperature averaged over the Great Plains; (b, c) summer heat wave frequency (HWF [days per summer]), and (d, e) heat wave amplitude (HWA [°C]) averaged over the (left) 1930s and (right) 1950s decade; (f) the summer HWA over the Great Plains, following the 10 driest (orange, average red) and wettest springs (light blue, average dark blue) respectively, calculated from two separate drought indices (standardized precipitation index [SPI], and palmer drought severity index [PDSI]), taken from all springs and summers over 1920–2012 and compared to a 5–95% range for an average of 1000 sets of 10 randomly sampled springs. The heat wave amplitude in summers following the driest springs is significantly larger than those following random springs. TXx and TNx values are taken from HadEX2 (1901–2010) and GHCNDEX (1951–2016). (Based on data from Cowan et al. (2017))

In the 1930s, concurrent with the Arctic warming, North America experienced a decadal scale climatic anomaly known as the “Dust Bowl droughts” which were accompanied by intense heat waves (see Figure 9). These followed prior wetter years, including the Great Mississippi flood of 1927 (Barry, 1997).

During the 1930s the Midwestern United States (including southern Canada) suffered a decade of drought and intense dust storms that destroyed farmland (Worster, 1979), contributing, along with the “Great Depression,” to the migration of farmers that is described in the novel “*Grapes of Wrath*” by John Steinbeck. Negative precipitation anomalies covered the entire central belt of the United States and reached to the Canadian Plains and to the Pacific Northwest during the 1930s (Schubert et al., 2004b; Schubert, Suarez, Pegion, Koster, & Bacmeister, 2004a); although larger ones are reconstructed for periods early in the Last Millennium (Woodhouse & Overpeck, 1998).

**Figure 10:**
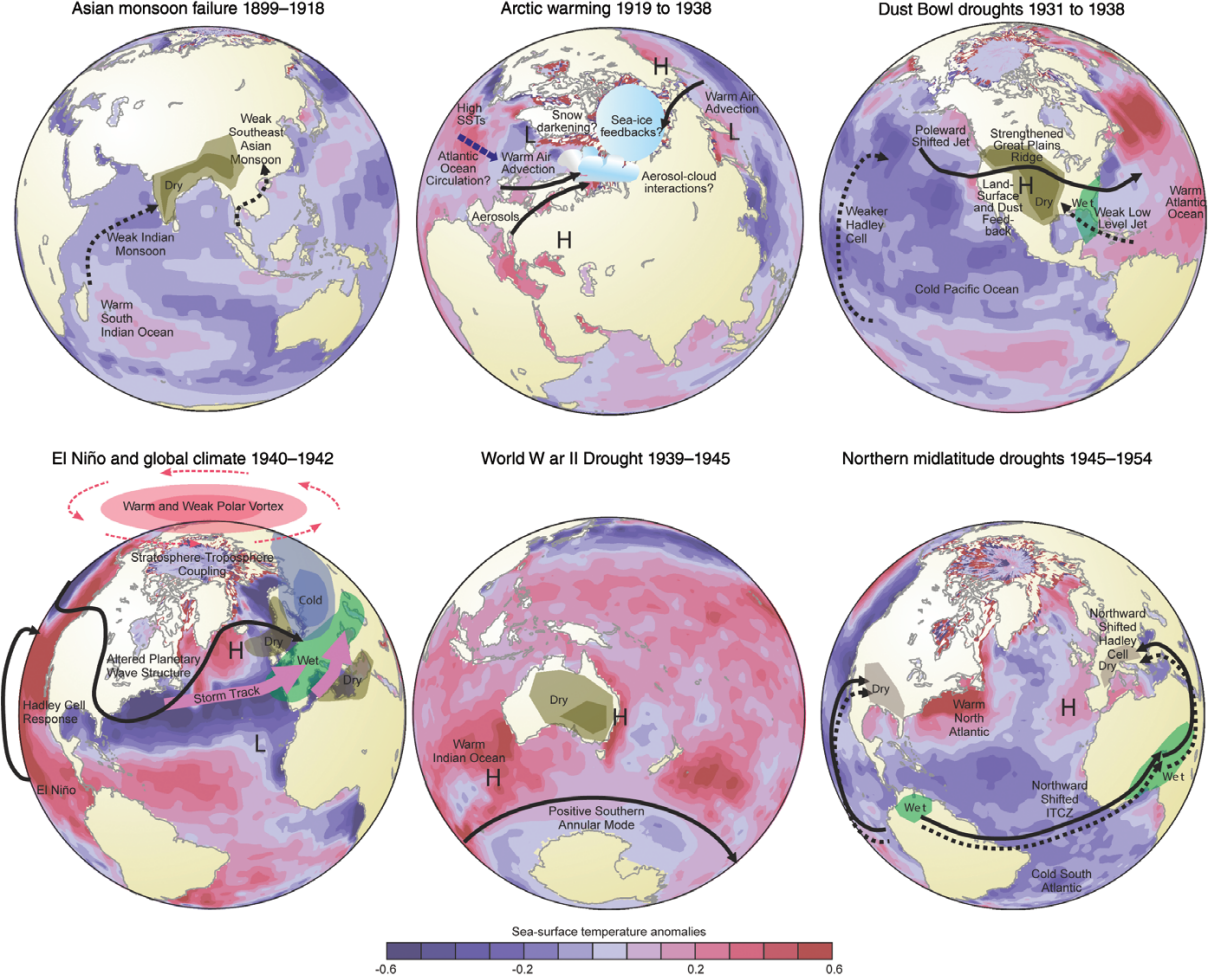
Schematic figure of six climatic anomalies during the ETCW. SST anomalies from HadSST1.1, given as annual mean over indicated period relative to the average of the preceding 15 and following 15 years. Mechanisms discussed in the text are shown schematically

Atmospheric simulations driven with observed SSTs typically reproduce drought conditions over the southern United States, although the location and intensity may vary (Brönnimann, Stickler, Griesser, Ewen, et al., 2009; Brönnimann, Stickler, Griesser, Fischer, et al., 2009; Cook et al., 2009; Cook, Miller, & Seager, 2008; Schubert et al., 2004a, 2004b; Seager, 2007; Seager, Kushnir, Herweijer, Naik, & Velez, 2005) and both tropical and extratropical SST anomalies are needed to reproduce the pattern. Additionally, these papers suggest that many atmospheric features are reproduced, such as a decreased southerly inflow of moist air (in accordance with a weakening of the Great Plains Low Level Jet), a continental‐wide mid‐tropospheric ridge, and a 200 hPa geopotential height anomaly pattern similar as in the observation‐based data. Observed SSTs at the time comprise a cooler tropical and North Pacific compared to preceding and following years, concurring with a warm tropical and North Atlantic (Schubert et al., 2004b; Seager et al., 2008; Figure 10) which may both have contributed to the establishment of a circulation pattern that caused a precipitation deficit in the Great Plains.

However, SSTs alone do not suffice as an explanation. Without land‐surface coupling an atmospheric model did not reproduce a strong drought in their model simulations (Schubert et al., 2004b), while the inclusion of land degradation (i.e., bare soil) and dust made the simulated drought much more realistic in terms of warmer temperature patterns and greater precipitation deficits (Cook et al., 2009). Rapid land cover changes prior to the 1930s contributed to new agricultural land being vulnerable to drought and erosion. Land–atmosphere interactions, such as reduced evaporation contributing to a precipitation deficit, and dust forcing heating and stabilizing the atmosphere may have amplified the droughts (Brönnimann, Stickler, Griesser, Ewen, et al., 2009; Brönnimann, Stickler, Griesser, Fischer, et al., 2009; Cook et al., 2008, 2009; Cook, Seager, & Miller, 2011; Schubert et al., 2004b; Seager et al., 2005, 2008).

The droughts were also accompanied by extreme heatwaves, with heat records set during the peak years of 1934 and 1936 still standing at the time of writing (Figure 9a). Given record temperatures, set over a large fraction of the United States (Abatzoglou & Barbero, 2014), were measured for both daily maxima and minima this was unlikely due to exposure changes or instrumentation biases but a real phenomenon. Heat waves during the 1930s were more frequent and intense than those during the 1950s drought decade (see Figure 9b–e). In general, heat waves over the North American Great Plains are closely linked to drought (Cowan et al., 2017); that is, heat waves following dry springs tend to be significantly earlier, hotter, and more frequent than those following average or wet springs (Figure 9f; note that heat wave frequency (HWF) describes the total number of heat wave days per season, whereas heat wave amplitude (HWA), is the anomaly of the hottest day of the hottest seasonal heat wave) consistent with findings for other regions (Hirschi et al., 2010; Mueller & Seneviratne, 2012). Dry springs were hence an important factor during the hottest Dust Bowl summers of 1934 and 1936 (Cowan et al., 2017). However, the role of SSTs in spring drought specifically is less than clear (Donat et al., 2016), as are the driving mechanisms of high decadal heat wave activity overall.

Could such heat waves occur again, possibly stronger due to global warming? In order to better understand and quantify the risk of recurrence, or strengthening, of such the damaging heat and drought events we need to better quantify the contribution by SSTs, random and unpredictable atmospheric variability, land surface feedbacks, and land management.

### 7.4 Global climate anomalies during the 1939–1942 El Niño

The “Dust Bowl” droughts ended with the onset of a strong El Niño event in the tropical Pacific in 1939/1940. El Niño conditions then predominated for the next 3 years. During this period, global climate anomalies emerged, including cold winters in Europe (see below), warm winters in Alaska, wet springs in central Europe, a drought in Australia and warm conditions in West Antarctica (Steig et al., 2013). In the stratosphere, total column ozone was extremely high over Europe, the Arctic, North America, and East Asia.

Many of these observed climatic anomalies (including the change in ozone and the cold winters in Europe) could have been contributed to by the strong, long‐lasting El Niño event (Brönnimann et al., 2004) combined with negative NAO conditions (which could have been made more likely by El Niño's imprint in the North Pacific [Li & Lau, 2012]). Planetary wave activity appeared to have increased, weakening the stratospheric polar vortex and increasing poleward transport of ozone.

Because of World War II, and changes in observational coverage and observational practice (in particular SSTs), the data uncertainty is unfortunately rather large in these years. Nevertheless, a recent experimental reanalysis incorporating surface and upper‐air data (Hersbach et al., 2017) confirmed the anomalies in atmospheric circulation.

Many of the circulation features are also successfully modeled in atmospheric model simulations driven by SSTs. The strong but southward shifted Hadley cell and positive PNA mode are well reproduced, for instance, in ERA‐20CM (Figure S7). Models typically also produce cold winters in Europe (Brönnimann et al., 2004), although the effect competes with strong interannual variability (Kenyon & Hegerl, 2008) and the magnitude in simulations is much smaller than in the observations, implying that internal atmospheric variability contributed significantly to the cold winters in Europe.

### 7.5 The World War II drought in Australia

Also during the late 1930s and early 1940s drought conditions affected Australia. Dry conditions started in 1937 and worsening during the subsequent years. In January 1939, heat waves and bushfires (known as “Black Friday” fires) had devastating effects on eastern Australia. Drought then worsened during the 1939–1942 El Niño event and persisted through to 1945.

Drought conditions in Australia are generally influenced by ENSO, Pacific Decadal Variability, the Indian Ocean Dipole Mode, and Southern Annular Mode (SAM; Verdon‐Kidd & Kiem, 2009; see Figure S7). The strong and extended El Niño event over 1939–1942 might have contributed to worsening the drought conditions, although the World War II drought started and ended during La Niña conditions, whose effect on precipitation might have been offset by the positive PDO (Cai et al., 2010) and by a positive SAM (Verdon‐Kidd & Kiem, 2009; note, however, that the SAM indices are very uncertain in these years). A positive SAM is generally associated with a southward shift in cold weather fronts, associated with high precipitation during the austral winter months, leading to drier conditions over southeast Australia (Risbey, Pook, McIntosh, Wheeler, & Hendon, 2009). Also, relative to the southern Indian Ocean, SSTs northwest of Australia were anomalously cool, depriving southeastern Australian of a tropical moisture source as is often the case during positive Indian Ocean dipole events (Ummenhofer et al., 2009).

### 7.6 Drought and hot summers combined with cold winters in Europe in the 1940s

The end of the ETCW is marked by drought conditions in central and southern Europe. Because economies had not yet recovered from World War II, the drought years (most notably 1945 and 1947) had large impacts in Germany and other European countries. The droughts coincide with a peak in the AMO phase (Sutton & Hodson, 2005; Figure 4). Atmospheric models reproduce major multidecadal changes in precipitation and temperature over Europe when driven by SSTs (Sutton & Hodson, 2005), although with unclear signal‐to‐noise ratio due to high atmospheric variability. Simulations also reproduce a tendency toward decreased precipitation in south‐central Europe and an increased frequency of summer anticyclonic situations in the 1940s (Brönnimann, 2015a). This was also a period of anomalously cold winters, during World War II, linked to anomalously high pressure over the Atlantic/European sector (see Figures 8, S8, and S9).

The peak of the positive AMO phase was accompanied by the most poleward position of the northern Hadley cell observed during the 20th century (Figure 7). Central Europe was thus more frequently under the influence of anticyclonic weather (Brönnimann et al., 2015), leading to summer heatwaves such as the 1947 heat wave which set Swiss records prior to 2003 (Schär et al., 2004).

The ETCW period ended in the 1940s (Figure 1). Subsequently, the Hadley cell moved equatorward (Figure 7) and the Northern Hemisphere (and the Arctic) cooled. During the subsequent decades, warming continued in the Southern Hemisphere, while the Northern Hemisphere cooled into the late 1970s (Hansen, Ruedy, Sato, & Lo, 2010). While anthropogenic aerosols have been suggested to be responsible for a large fraction of the cooling, internal variability also might have contributed (see Bindoff et al., 2013).

## 8 CONCLUSION

Longer records including recently digitized old records, together with reanalyses and large ensembles covering the entire 20th century provide new and important insight into the climatic anomalies during the early 20th century. These are not only interesting historically, but provide important evidence for events and anomalies that can occur and challenge climate models to explain or at least, sample them. Key events and anomalies include anomalously poor monsoon years around the turn of the century, the rapid Arctic warming into the 1920s, the Dust Bowl drought and heat waves in North America in the 1930s, and, approximately coincident with World War II, drought in Australia, and cold winters and hot summers in Europe in the 1940s (see overview schematic in Figure 10). These anomalous events occurred during a period of strong global‐scale warming, which can be attributed to a combination of external forcing (particularly, greenhouse gas increases, combined with a hiatus in volcanic events) and internal decadal variability. The exact contribution of each factor to large‐scale warming remains uncertain, largely due to uncertainty in the role of aerosols in the cooling or stabilization of climate following the middle of the 20th century.

## CONFLICT OF INTEREST

The authors have declared no conflicts of interest for this article.

## RELATED WIREs ARTICLES

https://doi.org/10.1002/wcc.18

https://doi.org/10.1002/wcc.21

https://doi.org/10.1002/wcc.380

https://doi.org/10.1002/wcc.121

## Supporting information

Appendix S1.

## References

[wcc522-bib-0001] Abatzoglou, J. T. , & Barbero, R. (2014). Observed and projected changes in absolute temperature records across the contiguous United States. Geophysical Research Letters, 41, 6501–6508. https://doi.org/10.1002/2014GL061441

[wcc522-bib-0002] Abram, N. J. , McGregor, H. , Tierney, J. E. , Evans, M. N. , McKay, N. P. , Kaufman, D. S. , & PAGES 2k Consortium . (2016). Early onset of industrial‐era warming across the oceans and continents. Nature, 536, 411–418. https://doi.org/10.1038/nature19082 27558063 10.1038/nature19082

[wcc522-bib-0003] Allan, R. , & Ansell, T. (2006). A New globally complete monthly historical gridded mean sea level pressure dataset (HadSLP2): 1850–2004. Journal of Climate, 19, 5816–5842.

[wcc522-bib-0004] Allan, R. , Brohan, P. , Compo, G. , Stone, R. , Luterbacher, J. , & Brönnimann, S. (2011). The International Atmospheric Circulation Reconstructions over the Earth (ACRE) initiative. Bulletin of the American Meteorological Society, 92, 1421–1425. https://doi.org/10.1175/2011BAMS3218.1

[wcc522-bib-0005] Anderson, D. M. , Mauk, E. M. , Wahl, E. R. , Morrill, C. , Wagner, A. J. , Easterling, D. , & Rutishauser, T. (2013). Global warming in an independent record of the past 130 years. Geophysical Research Letters, 40, 189–193. https://doi.org/10.1029/2012GL054271

[wcc522-bib-0006] Auchmann, R. , & Brönnimann, S. (2012). A physics‐based correction model for homogenizing sub‐daily temperature series. Journal of Geophysical Research, 117, D17119. https://doi.org/10.1029/2012JD018067

[wcc522-bib-0007] Barry, J. M. (1997). Rising tide: The great Mississippi flood of 1927 and how it changed America. New York, NY: Simon & Schuster.

[wcc522-bib-0008] Beitsch, A. , Jungclaus, J. H. , & Zanchettin, D. (2014). Patterns of decadal‐scale Arctic warming events in simulated climate. Climate Dynamics, 43, 1773–1789. https://doi.org/10.1007/s00382-013-2004-5

[wcc522-bib-0009] Bindoff, N. L. , Stott, P. A. , AchutaRao, K. M. , Allen, M. R. , Gillett, N. , Gutzler, D. , … Zhang, X. (2013). Detection and attribution of climate change: From global to regional In Stocker T. F., Qin D., Plattner G.‐K., Tignor M., Allen S. K., Boschung J., et al. (Eds.), Climate change 2013: The physical science basis. Contribution of working group I to the fifth assessment report of the intergovernmental panel on climate change (pp. 867–952). Cambridge, England; New York, NY: Cambridge University Press http://doi.org/10.1017/CBO9781107415324.022

[wcc522-bib-0010] Birkeland, B. (1930). Temperaturvariationen auf Spitzbergen. Meteorologische Zeitschrift, 47, 234–236.

[wcc522-bib-0011] Booth, B. B. B. , Dunstone, N. J. , Halloran, P. R. , Andrews, T. , & Bellouin, N. (2012). Aerosols implicated as a prime driver of twentieth‐century North Atlantic climate variability. Nature, 484(7393), 228–232. https://doi.org/10.1038/nature10946 22498628 10.1038/nature10946

[wcc522-bib-0012] Brönnimann, S. (2009). Early twentieth‐century warming. Nature Geoscience, 2(11), 735–736. https://doi.org/10.1038/ngeo670

[wcc522-bib-0013] Brönnimann, S. (2015a). Climatic changes since 1700 In Advances in global change research (Vol. 55, p. 360). Cham and Heidelberg, Germany; New York, NY; Dordrecht and London, England: Springer http://doi.org/10.1007/978-3-319-19042-6

[wcc522-bib-0014] Brönnimann, S. (2015b). Pacemakers of warming. Nature Geoscience, 8, 87–89. https://doi.org/10.1038/ngeo2330

[wcc522-bib-0015] Brönnimann, S. , Fischer, A. , Rozanov, E. , Poli, P. , Compo, G. , & Sardeshmukh, P. (2015). Southward shift of the northern tropical belt from 1945 to 1980. Nature Geoscience, 8, 969–974. https://doi.org/10.1038/NGEO2568

[wcc522-bib-0016] Brönnimann, S. , Grant, A. , Compo, G. , Ewen, T. , Griesser, T. , Fischer, A. M. , … Stickler, A. (2012). A multi‐data set comparison of the vertical structure of temperature variability and change over the Arctic during the past 100 years. Climate Dynamics, 39, 1577–1598. https://doi.org/10.1007/s00382-012-1291-6

[wcc522-bib-0017] Brönnimann, S. , & Krämer, D. (2016). Tambora and the “year without a summer” of 1816. Geographica Bernensia, G90, 48.

[wcc522-bib-0018] Brönnimann, S. , Luterbacher, J. , Staehelin, J. , Svendby, T. , Hansen, G. , & Svenøe, T. (2004). Extreme climate of the global troposphere and stratosphere in 1940–42 related to El Niño. Nature, 431, 971–974. https://doi.org/10.1038/nature02933.1 15496919 10.1038/nature02982

[wcc522-bib-0019] Brönnimann, S. , Stickler, A. , Griesser, T. , Ewen, T. , Grant, A. N. , Fischer, A. M. , … Ross, T. (2009). Exceptional atmospheric circulation during the “Dust Bowl.” Geophysical Research Letters, 36(8), 1–6. https://doi.org/10.1029/2009GL037612

[wcc522-bib-0020] Brönnimann, S. , Stickler, A. , Griesser, T. , Fischer, A. , Grant, A. , Ewen, T. , … Peter, T. (2009). Variability of large‐scale atmospheric circulation indices for the northern hemisphere during the past 100 years. Meteorologische Zeitschrift, 18(4), 379–396. https://doi.org/10.1127/0941-2948/2009/0389

[wcc522-bib-0021] Brunet, M. , Asin, J. , Sigró, J. , Bañón, M. , García, F. , Aguilar, E. , … Jones, P. (2011). The minimization of the screen bias from ancient Western Mediterranean air temperature records: An exploratory statistical analysis. International Journal of Climatology, 31, 1879–1895. https://doi.org/10.1002/joc.2192

[wcc522-bib-0022] Cai, W. , van Rensch, P. , Cowan, T. , & Sullivan, A. (2010). Asymmetry in ENSO teleconnection with regional rainfall, its multidecadal variability, and impact. Journal of Climate, 23(18), 4944–4955. https://doi.org/10.1175/2010JCLI3501.1

[wcc522-bib-0023] Callendar, G. (1938). The artificial production of carbon dioxide and its influence on temperature. Quarterly Journal of the Royal Meteorological Society, 64, 223–240.

[wcc522-bib-0024] Chan, D. , & Huybers, P. (n.d.). *Homogeneous early 20th century sea surface warming after correcting for historical artifacts.* Manuscript submitted for publication.

[wcc522-bib-0025] Chen, X. , & Tung, K. (2014). Varying planetary heat sink led to global‐warming slowdown and acceleration. Science, 345, 897–903. https://doi.org/10.1126/science.1254937 25146282 10.1126/science.1254937

[wcc522-bib-0026] Chylek, P. , Folland, C. K. , Lesins, G. , Dubey, M. K. , & Wang, M. (2009). Arctic air temperature change amplification and the Atlantic Multidecadal Oscillation. Geophysical Research Letters, 36, L14801 https://doi.org/10.1029/2009GL038777

[wcc522-bib-0027] Clement, A. , Bellomo, K. , Murphy, L. N. , Cane, M. A. , Mauritsen, T. , Rädel, G. , & Stevens, B. (2015). The Atlantic Multidecadal Oscillation without a role for ocean circulation. Science, 350(6258), 320–324. https://doi.org/10.1126/science.aab3980 26472908 10.1126/science.aab3980

[wcc522-bib-0028] Coddington, O. , Lean, J. , Pilewskie, P. , Snow, M. , & Lindholm, D. (2016). A solar irradiance climate data record. Bulletin of the American Meteorological Society, 97, 1265–1282. https://doi.org/10.1175/BAMS-D-14-00265.1

[wcc522-bib-0029] Compo, G. P. , Whitaker, J. S. , Sardeshmukh, P. D. , Matsui, N. , Allan, R. J. , Yin, X. , … Worley, S. J. (2011). The twentieth century reanalysis project. Quarterly Journal of the Royal Meteorological Society, 137(654), 1–28. https://doi.org/10.1002/qj.776

[wcc522-bib-0030] Cook, B. I. , Miller, R. L. , & Seager, R. (2008). Dust and sea surface temperature forcing of the 1930s “Dust Bowl” drought. Geophysical Research Letters, 35, L08710 https://doi.org/10.1029/2008GL033486

[wcc522-bib-0031] Cook, B. I. , Miller, R. L. , & Seager, R. (2009). Amplification of the North American “Dust Bowl” drought through human‐induced land degradation. Proceedings of the National Academy of Sciences of the United States of America, 106(13), 4997–5001. https://doi.org/10.1073/pnas.0810200106 19289836 10.1073/pnas.0810200106 PMC2664008

[wcc522-bib-0032] Cook, B. I. , Seager, R. , & Miller, R. L. (2011). Atmospheric circulation anomalies during two persistent north american droughts: 1932–1939 and 1948–1957. Climate Dynamics, 36(11–12), 2339–2355. https://doi.org/10.1007/s00382-010-0807-1

[wcc522-bib-0034] Cowan, T. , Hegerl, G. C. , Colfescu, I. , Bollasina, M. A. , Purich, A. , & Boschat, G. (2017). Factors contributing to record‐breaking heat waves over the great plains during the 1930s Dust Bowl. Journal of Climate, 30, 2437–2461. https://doi.org/10.1175/JCLI-D-16-0436.1

[wcc522-bib-0035] Cowtan, K. , Hausfather, Z. , Hawkins, E. , Jacobs, P. , Mann, M. E. , Miller, S. K. , … Way, R. G. (2015). Robust comparison of climate models with observations using blended land air and ocean sea surface temperatures. Geophysical Research Letters, 42, 6526–6534. https://doi.org/10.1002/2015GL064888

[wcc522-bib-0036] Cowtan, K. , & Way, R. G. (2014). Coverage bias in the HadCRUT4 temperature series and its impact on recent temperature trends. Quarterly Journal of the Royal Meteorological Society, 140, 1935–1944. https://doi.org/10.1002/qj.2297

[wcc522-bib-0037] Crook, J. A. , & Forster, P. M. (2011). A balance between radiative forcing and climate feedback in the modeled 20th century temperature response. Journal of Geophysical Research, 116, D17108 https://doi.org/10.1029/2011JD015924

[wcc522-bib-0038] Crowley, T. J. , Obrochta, S. P. , & Liu, J. (2014). Recent global temperature “plateau” in the context of a new proxy. Earth's Future, 2, 281–294. https://doi.org/10.1002/2013EF000216

[wcc522-bib-0039] Delworth, T. L. , & Knutson, T. R. (2000). Simulation of early 20th century global warming. Science, 287, 2246–2250. https://doi.org/10.1126/science.287.5461.2246 10731143 10.1126/science.287.5461.2246

[wcc522-bib-0040] Donat, M. G. , King, A. D. , Overpeck, J. T. , Alexander, L. V. , Durre, I. , & Karoly, D. J. (2016). Extraordinary heat during the 1930s US Dust Bowl and associated large‐scale conditions. Climate Dynamics, 46, 413–426. https://doi.org/10.1007/s00382-015-2590-5

[wcc522-bib-0041] Drijfhout, S. S. , Blaker, A. T. , Josey, S. A. , Nurser, A. J. G. , Sinha, B. , & Balmaseda, M. A. (2014). Surface warming hiatus caused by increased heat uptake across multiple ocean basins. Geophysical Research Letters, 41, 7868–7874. https://doi.org/10.1002/2014GL061456

[wcc522-bib-0042] England, M. H. , McGregor, S. , Spence, P. , Meehl, G. A. , Timmermann, A. , Cai, W. , … Santoso, A. (2014). Recent intensification of wind‐driven circulation in the Pacific and the ongoing warming hiatus. Nature Climate Change, 4, 222–227. https://doi.org/10.1038/nclimate2106

[wcc522-bib-0043] Ewen, T. , Brönnimann, S. , & Annis, J. (2008). An Extended Pacific–North American index from upper‐air historical data back to 1922. Journal of Climate, 21, 1295–1308. https://doi.org/10.1175/2007JCLI1951.1

[wcc522-bib-0044] Faurschou Knudsen, M. , Jacobsen, B. H. , Seidenkrantz, M.‐S. , & Olsen, J. (2014). Evidence for external forcing of the Atlantic Multidecadal Oscillation since termination of the Little Ice Age. Nature Communications, 5, 1–8. https://doi.org/10.1038/ncomms4323 10.1038/ncomms4323 PMC3948066 24567051

[wcc522-bib-0045] Flato, G. , Marotzke, J. , Abiodun, B. , Braconnot, P. , Chou, S. C. , Collins, W. , … Rummukainen, M. (2013). Evaluation of climate models In Stocker T. F., Qin D., Plattner G.‐K., Tignor M., Allen S. K., Boschung J., et al. (Eds.), Climate change 2013: The physical science basis. Contribution of working group I to the fifth assessment report of the intergovernmental panel on climate change. Cambridge, England; New York, NY: Cambridge University Press.

[wcc522-bib-0046] Fyfe, J. C. , Meehl, G. A. , England, M. H. , Mann, M. E. , Santer, B. D. , Flato, G. M. , … Swart, N. C. (2016). Making sense of the early‐2000s warming slowdown. Nature Climate Change, 6(3), 224–228. https://doi.org/10.1038/nclimate2938

[wcc522-bib-0047] Fyfe, J. C. , Von Salzen, K. , Gillett, N. P. , Arora, V. K. , Flato, G. M. , & McConnell, J. R. (2013). One hundred years of Arctic surface temperature variation due to anthropogenic influence. Scientific Reports, 3, 2645 https://doi.org/10.1038/srep02645 10.1038/srep02645 PMC3770965 24025852

[wcc522-bib-0048] Gastineau, G. , & Frankignoul, C. (2015). Influence of the North Atlantic SST variability on the atmospheric circulation during the twentieth century. Journal of Climate, 28, 1396–1416. https://doi.org/10.1175/JCLI-D-14-00424.1

[wcc522-bib-0049] Giese, B. S. , Compo, G. , Slowey, N. , Sardeshmukh, P. , Carton, J. , Ray, S. , & Whitaker, J. (2010). The 1918/19 El Niño. Bulletin of the American Meteorological Society, 91, 177–183. https://doi.org/10.1175/2009BAMS2903.1

[wcc522-bib-0050] Gillett, N. , Arora, V. , Matthews, D. , & Allen, M. (2013). Constraining the ratio of global warming to cumulative CO_2_ emissions using CMIP5 simulations. Journal of Climate, 26(2009), 6844–6858. https://doi.org/10.1175/JCLI-D-12-00476.1

[wcc522-bib-0051] Goswami, B. N. , Madhusoodanan, M. S. , Neema, C. P. , & Sengupta, D. (2006). A physical mechanism for North Atlantic SST influence on the Indian summer monsoon. Geophysical Research Letters, 33, L02706 https://doi.org/10.1029/2005GL024803

[wcc522-bib-0052] Grant, A. , Brönnimann, S. , Ewen, T. , Griesser, T. , & Stickler, A. (2009). The early twentieth century warm period in the European Arctic. Meteorologische Zeitschrift, 18(4), 425–432. https://doi.org/10.1127/0941-2948/2009/0391

[wcc522-bib-0053] Grant, A. , Brönnimann, S. , & Haimberger, L. (2008). Recent Arctic warming vertical structure contested. Nature, 455, E2–E3. https://doi.org/10.1038/nature07257 18784660 10.1038/nature07257

[wcc522-bib-0054] Gray, L. J. , Beer, J. , Geller, M. , Haigh, J. D. , Lockwood, M. , Matthes, K. , … White, W. (2010). Solar influence on climate. Reviews of Geophysics, 48, RG4001 https://doi.org/10.1029/2009RG000282

[wcc522-bib-0055] Gregory, J. M. (2010). Long‐term effect of volcanic forcing on ocean heat content. Geophysical Research Letters, 37, L22701 https://doi.org/10.1029/2010GL045507

[wcc522-bib-0056] Häkkinen, S. , Rhines, P. , & Worthen, D. (2011). Atmospheric blocking and Atlantic multidecadal ocean variability. Science, 334(6056), 655–660. https://doi.org/10.1126/science.1205683 22053046 10.1126/science.1205683

[wcc522-bib-0057] Hansen, J. , Ruedy, R. , Sato, M. , & Lo, K. (2010). Global surface temperature change. Reviews of Geophysics, 48, RG4004 https://doi.org/10.1029/2010RG000345

[wcc522-bib-0058] Hartmann, D. L. , Klein Tank, A. M. G. , Rusticucci, M. , Alexander, L. V. , Brönnimann, S. , Charabi, Y. , … Zhai, P. M. (2013). Observations: Atmosphere and surface In Stocker T. F., Qin D., Plattner G.‐K., Tignor M., Allen S. K., Boschung J., et al. (Eds.), Climate change 2013: The physical science basis. Contribution of working group I to the fifth assessment report of the intergovernmental panel on climate change (pp. 159–254). Cambridge, England; New York, NY: Cambridge University Press.

[wcc522-bib-0059] Hasselmann, K. (1976). Stochastic climate models Part I. Theory. Tellus, 28, 473–485. https://doi.org/10.1111/j.2153-3490.1976.tb00696.x

[wcc522-bib-0060] Hedemann, C. , Mauritsen, T. , Jungclaus, J. , & Marotzke, J. (2017). The subtle origins of surface‐warming hiatuses. Nature Climate Change, 7, 336–339. https://doi.org/10.1038/NCLIMATE3274

[wcc522-bib-0061] Hegerl, G. , Black, E. , Allan, R. , Ingram, W. , Polson, D. , Trenberth, K. , … Zhang, X. (2015). Challenges in quantifying changes in the global water cycle. Bulletin of the American Meteorological Society, 96, 1097–1115. https://doi.org/10.1175/BAMS-D-13-00212.1

[wcc522-bib-0062] Hegerl, G. , Crowley, T. , Allen, M. , Hyde, W. , Pollack, H. , Smerdon, J. , & Zorita, E. (2007). Detection of human influence on a new, validated 1500‐year temperature reconstruction. Journal of Climate, 20, 650–666. https://doi.org/10.1175/JCLI4011.1

[wcc522-bib-0063] Hegerl, G. , & Zwiers, F. (2011). Use of models in detection and attribution of climate change. WIREs Climate Change, 2(4), 570–591. https://doi.org/10.1002/wcc.121

[wcc522-bib-0064] Hegerl, G. C. , Crowley, T. J. , Baum, S. K. , Kim, K. , & Hyde, W. T. (2003). Detection of volcanic, solar and greenhouse gas signals in paleo‐reconstructions of Northern Hemispheric temperature. Geophysical Research Letters, 30(5), 1242 https://doi.org/10.1029/2002GL016635

[wcc522-bib-0065] Hegerl, G. C. , Hasselmann, K. , Cubasch, U. , Mitchell, J. F. B. , Roeckner, E. , Voss, R. , & Waszkewitz, J. (1997). Multi‐fingerprint detection and attribution analysis of greenhouse gas, greenhouse gas‐plus‐aerosol and solar forced climate change. Climate Dynamics, 13, 613–634.

[wcc522-bib-0066] Hegerl, G. C. , von Storch, H. , Hasselmann, K. , Santer, B. D. , Cubasch, U. , & Jones, P. D. (1996). Detecting greenhouse gas induced climate change with an optimal fingerprint method. Journal of Climate, 9, 2281–2306.

[wcc522-bib-0067] Hersbach, H. , Brönnimann, S. , Haimberger, L. , Mayer, M. , Villiger, L. , Comeaux, J. , … Worley, S. J. (2017). The potential value of early (1939–1967) upper‐air data in atmospheric climate reanalysis. Quarterly Journal of the Royal Meteorological Society, 143, 1197–1210. https://doi.org/10.1002/qj.3040

[wcc522-bib-0068] Hersbach, H. , Peubey, C. , Simmons, A. , Berrisford, P. , Poli, P. , & Dee, D. (2015). ERA‐20CM: A twentieth‐century atmospheric model ensemble. Quarterly Journal of the Royal Meteorological Society, 141(July), 2350–2375. https://doi.org/10.1002/qj.2528

[wcc522-bib-0069] Hirschi, M. , Seneviratne, S. I. , Alexandrov, V. , Boberg, F. , Boroneant, C. , Christensen, O. B. , … Stepanek, P. (2010). Observational evidence for soil‐moisture impact on hot extremes in southeastern Europe. Nature Geoscience, 4, 17–21. https://doi.org/10.1038/ngeo1032

[wcc522-bib-0070] Hurrell, J. W. (1995). Decadal trends in the North Atlantic Oscillation: Regional temperatures and precipitation. Science, 269, 676–679. https://doi.org/10.1126/science.269.5224.676 17758812 10.1126/science.269.5224.676

[wcc522-bib-0071] Iles, C. , & Hegerl, G. (2017). Role of the North Atlantic Oscillation in decadal temperature trends. Environmental Research Letters, 12, 114010 https://doi.org/10.1088/1748-9326/aa9152

[wcc522-bib-0072] Jones, J. , Fogt, R. , Widmann, M. , Marshall, G. , Jones, P. , & Visbeck, M. (2009). Historical SAM variability. Part I: Century‐length seasonal reconstructions. Journal of Climate, 22, 5319–5345. https://doi.org/10.1175/2009JCLI2785.1

[wcc522-bib-0073] Karl, T. , Arguez, A. , Huang, B. , Lawrimore, J. , McMahon, J. , Menne, M. , … Zhang, H. (2015). Possible artifacts of data biases in the recent global surface warming hiatus. Science, 348, 1469–1472. https://doi.org/10.1126/science.aaa5632 26044301 10.1126/science.aaa5632

[wcc522-bib-0074] Kennedy, J. J. , Rayner, N. A. , Smith, R. O. , Parker, D. E. , & Saunby, M. (2011). Reassessing biases and other uncertainties in sea surface temperature observations measured in situ since 1850: 1. Measurement and sampling uncertainties. Journal of Geophysical Research, 116, D14103 https://doi.org/10.1029/2010JD015218

[wcc522-bib-0075] Kenyon, J. , & Hegerl, G. (2008). Influence of modes of climate variability on global temperature extremes. Journal of Climate, 21, 3872–3889. https://doi.org/10.1175/2008JCLI2125.1

[wcc522-bib-0076] Knight, J. R. (2009). The Atlantic Multidecadal Oscillation inferred from the forced climate response in coupled general circulation models. Journal of Climate, 22, 1610–1625. https://doi.org/10.1175/2008JCLI2628.1

[wcc522-bib-0077] Knight, J. R. , Allan, R. J. , Folland, C. K. , Vellinga, M. , & Mann, M. E. (2005). A signature of persistent natural thermohaline circulation cycles in observed climate. Geophysical Research Letters, 32, L20708 https://doi.org/10.1029/2005GL024233

[wcc522-bib-0078] Krishna Kumar, K. , Rajagopalan, B. , Hoerling, M. , Bates, G. , & Cane, M. (2006). Unraveling the mystery of Indian monsoon failure during El Niño. Science, 314, 115–119. 16959975 10.1126/science.1131152

[wcc522-bib-0079] Krishnamurthy, L. , & Krishnamurthy, V. (2016). Teleconnections of Indian monsoon rainfall with AMO and Atlantic tripole. Climate Dynamics, 46(7), 2269–2285. https://doi.org/10.1007/s00382-015-2701-3

[wcc522-bib-0080] Lee, S. , Park, W. , Baringer, M. O. , Gordon, A. L. , Huber, B. , & Liu, Y. (2015). Pacific origin of the abrupt increase in Indian Ocean heat content during the warming hiatus. Nature Geoscience, 8(J), 445–449. https://doi.org/10.1038/NGEO2438

[wcc522-bib-0081] Li, Y. , & Lau, N. C. (2012). Impact of ENSO on the atmospheric variability over the North Atlantic in late winter—Role of transient eddies. Journal of Climate, 25, 320–342. https://doi.org/10.1175/JCLI-D-11-00037.1

[wcc522-bib-0082] Lockwood, M. , Harrison, R. G. , Woollings, T. , & Solanki, S. K. (2010). Are cold winters in Europe associated with low solar activity? Environmental Research Letters, 5, 024001 https://doi.org/10.1088/1748-9326/5/2/024001

[wcc522-bib-0083] Malik, A. , Brönnimann, S. , & Perona, P. (2017). Statistical link between external climate forcings and modes of ocean variability. Climate Dynamics, 50(9), 3649–3670. https://doi.org/10.1007/s00382-017-3832-5

[wcc522-bib-0084] Malik, A. , Brönnimann, S. , Stickler, A. , Raible, C. C. , Muthers, S. , Anet, J. , … Schmutz, W. (2017). Decadal to multi‐decadal scale variability of Indian summer monsoon rainfall in the coupled ocean‐atmosphere‐chemistry climate model SOCOL‐MPIOM. Climate Dynamics, 49, 3551–3572. https://doi.org/10.1007/s00382-017-3529-9

[wcc522-bib-0085] Mantua, N. J. , Hare, S. R. , Zhang, Y. , Wallace, J. M. , & Francis, R. C. (1997). A Pacific Interdecadal Climate Oscillation with impacts on salmon production. Bulletin of the American Meteorological Society, 78, 1069–1079. https://doi.org/10.1175/1520-0477(1997)078%3C1069:APICOW%3E2.0.CO;2

[wcc522-bib-0086] McCabe, G. J. , Palecki, M. A. , & Betancourt, J. L. (2004). Pacific and Atlantic Ocean influences on multidecadal drought frequency in the United States. Proceedings of the National Academy of Sciences, 101(12), 4136–4141. https://doi.org/10.1073/pnas.0306738101 10.1073/pnas.0306738101 PMC384707 15016919

[wcc522-bib-0087] McCarthy, G. D. , Smeed, D. A. , Johns, W. E. , Frajka‐Williams, E. , Moat, B. I. , Rayner, D. , … Bryden, H. L. (2015). Progress in oceanography measuring the Atlantic meridional overturning circulation at 26°N. Progress in Oceanography, 130, 91–111. https://doi.org/10.1016/j.pocean.2014.10.006

[wcc522-bib-0088] McConnell, J. , Edwards, R. , Kok, G. , Flanner, M. , Zender, C. , Saltzman, E. , … Kahl, J. (2007). 20th‐century industrial black carbon emissions altered Arctic climate forcing. Science, 317, 1381–1384. 17690261 10.1126/science.1144856

[wcc522-bib-0089] Medhaug, I. , Stolpe, M. B. , Fischer, E. M. , & Knutti, R. (2017). Reconciling controversies about the “global warming hiatus.” Nature, 545(7652), 41–47. https://doi.org/10.1038/nature22315 28470193 10.1038/nature22315

[wcc522-bib-0090] Meehl, G. A. , Arblaster, J. M. , Fasullo, J. T. , Hu, A. , & Trenberth, K. E. (2011). Model‐based evidence of deep‐ocean heat uptake during surface‐temperature hiatus periods. Nature Climate Change, 1(7), 360–364. https://doi.org/10.1038/nclimate1229

[wcc522-bib-0091] Morice, C. P. , Kennedy, J. J. , Rayner, N. A. , & Jones, P. D. (2012). Quantifying uncertainties in global and regional temperature change using an ensemble of observational estimates: The HadCRUT4 data set. Journal of Geophysical Research, 117, 1–22. https://doi.org/10.1029/2011JD017187

[wcc522-bib-0092] Mueller, B. , & Seneviratne, S. I. (2012). Hot days induced by precipitation deficits at the global scale. Proceedings of the National Academy of Sciences, 109(31), 12398–12403. https://doi.org/10.1073/pnas.1204330109 10.1073/pnas.1204330109 PMC3411978 22802672

[wcc522-bib-0093] Müller, W. , Matei, D. , Bersch, M. , Jungclaus, J. H. , Haak, H. , Lohmann, K. , … Marotzke, J. (2015). A twentieth‐century reanalysis forced ocean model to reconstruct the North Atlantic climate variation during the 1920s. Climate Dynamics, 1935–1955. https://doi.org/10.1007/s00382-014-2267-5

[wcc522-bib-0094] National Academies of Sciences, Engineering, & and Medicine . (2016). Attribution of extreme weather events in the context of climate change. Washington, DC: The National Academies Press https://doi.org/http://doi.org/10.17226/21852

[wcc522-bib-0095] Neely III, R. R. , & Schmidt, A . (2016). *VolcanEESM: Global volcanic sulphur dioxide (SO* _*2*_ *) emissions database from 1850 to present—Version 1.0. Centre for Environmental Data Analysis* http://dx.doi.org/10.5285/76ebdc0b-0eed-4f70-b89e-55e606bcd568

[wcc522-bib-0096] Newman, M. , Alexander, M. , Ault, T. , Cobb, K. , Deser, C. , Di Lorenzo, E. , … Smith, C. (2016). The Pacific Decadal Oscillation, revisited. Journal of Climate, 29, 4399–4427. https://doi.org/10.1175/JCLI-D-15-0508.1

[wcc522-bib-0097] Nordli, Ø. , Przybylak, R. , Ogilvie, A. , & Isaksen, K. (2014). Long‐term temperature trends and variability on Spitsbergen: The extended Svalbard airport temperature series, 1898 2012. Polar Research, 33, 21349 https://doi.org/10.3402/polar.v33.21349

[wcc522-bib-0098] Otterå, O. H. , Bentsen, M. , Drange, H. , & Suo, L. (2010). External forcing as a metronome for Atlantic multidecadal variability. Nature Geoscience, 3(10), 688–694. https://doi.org/10.1038/ngeo955

[wcc522-bib-0099] Overland, J. E. , & Wang, M. (2005). The third Arctic climate pattern: 1930s and early 2000s. Geophysical Research Letters, 32, L23808 https://doi.org/10.1029/2005GL024254

[wcc522-bib-0100] Parker, D. E. , Jones, P. D. , Folland, C. K. , & Bevan, A. (1994). Interdecadal changes of surface temperature since the late nineteenth century. Journal of Geophysical Research, 99, 14373–14399. https://doi.org/10.1029/94JD00548

[wcc522-bib-0101] Poli, P. , Hersbach, H. , Dee, D. , Berrisford, P. , Simmons, A. , Vitart, F. , … Fisher, M. (2016). ERA‐20C: An atmospheric reanalysis of the twentieth century. Journal of Climate, 29, 4083–4097. https://doi.org/10.1175/JCLI-D-15-0556.1

[wcc522-bib-0102] Polson, D. , Hegerl, G. C. , & Solomon, S. (2016). Precipitation sensitivity to warming estimated from long island records. Environmental Research Letters, 11, 074024 https://doi.org/10.1088/1748-9326/11/7/074024

[wcc522-bib-0103] Raible, C. C. , Brönnimann, S. , Auchmann, R. , Brohan, P. , Frölicher, T. L. , Graf, H. , … Wegmann, M. (2016). Tambora 1815 as a test case for high impact volcanic eruptions: Earth system effects. WIREs Climate Change, 7, 569–589. https://doi.org/10.1002/wcc.407 10.1002/wcc.407 PMC6686350 31423155

[wcc522-bib-0104] Ribes, A. , & Terray, L. (2013). Application of regularised optimal fingerprinting to attribution. Part II: Application to global near‐surface temperature. Climate Dynamics, 41, 2837–2853. https://doi.org/10.1007/s00382-013-1736-6

[wcc522-bib-0105] Ring, M. J. , Lindner, D. , Cross, E. F. , & Schlesinger, M. E. (2012). Causes of the global warming observed since the 19th century. Atmospheric and Climate Sciences, 2(4), 401–415. https://doi.org/10.4236/acs.2012.24035

[wcc522-bib-0106] Risbey, J. S. , Pook, M. J. , McIntosh, P. C. , Wheeler, M. C. , & Hendon, H. H. (2009). On the remote drivers of rainfall variability in Australia. Monthly Weather Review, 137(10), 3233–3253. https://doi.org/10.1175/2009MWR2861.1

[wcc522-bib-0107] Roberts, C. D. , Palmer, M. D. , McNeall, D. , & Collins, M. (2015). Quantifying the likelihood of a continued hiatus in global warming. Nature Climate Change, 5, 337–342. https://doi.org/10.1038/NCLIMATE2531

[wcc522-bib-0108] Rogers, J. (1985). Atmospheric circulation changes associated with the warming over the northern North Atlantic in the 1920s. Journal of Climate and Applied Meteorology, 24, 1303–1310.

[wcc522-bib-0109] Rohde, R. , Muller, R. , Jacobsen, R. , Perlmutter, S. , Rosenfeld, A. , Wurtele, J. , … Mosher, S. (2013). Berkeley Earth Temperature Averaging Process. Geoinformatics & Geostatistics: An Overview, 1, 2 http://doi.org/10.4172/2327-4581.1000103

[wcc522-bib-0110] Saji, N. H. , Goswami, B. N. , Vinayachandran, P. N. , & Yamagata, T. (1999). A dipole mode in the tropical Indian Ocean. Nature, 401, 360–363. https://doi.org/10.1038/43854 16862108 10.1038/43854

[wcc522-bib-0111] Scaife, A. , Kucharski, F. , Folland, C. , Kinter, J. , Brönnimann, S. , Fereday, D. , … Zhou, T. (2009). The CLIVAR C20C project: Selected twentieth century climate events. Climate Dynamics, 33, 603–614. https://doi.org/10.1007/s00382-008-0451-1

[wcc522-bib-0112] Schär, C. , Vidale, P. , Lüthi, D. , Frei, C. , Häberli, C. , Liniger, M. , & Appenzeller, C. (2004). The role of increasing temperature variability in European summer heatwaves. Nature, 427(January), 332–336. https://doi.org/10.1038/nature02230.1. 14716318 10.1038/nature02300

[wcc522-bib-0113] Scherhag, R. (1939a). Die Erwärmung des Polargebiets. Annalen der Hydrographie und maritimen Meteorologie, 67, 57–67.

[wcc522-bib-0114] Scherhag, R. (1939b). Die gegenwärtige Milderung der Winter und ihre Ursachen. Annalen der Hydrographie und maritimen Meteorologie, 67, 292–303.

[wcc522-bib-0115] Schlesinger, M. , & Ramankutty, N. (1994). An oscillation in the global climate system of period 65–70 years. Nature, 367, 723–726.

[wcc522-bib-0116] Schneider, U. , Becker, A. , Finger, P. , Meyer‐christoffer, A. , Ziese, M. , & Rudolf, B. (2014). GPCC's new land surface precipitation climatology based on quality‐controlled in situ data and its role in quantifying the global water cycle. Theoretical Applied Climatology, 115, 15–40. https://doi.org/10.1007/s00704-013-0860-x

[wcc522-bib-0117] Schubert, S. D. , Suarez, M. J. , Pegion, P. J. , Koster, R. D. , & Bacmeister, J. T. (2004a). Causes of long‐term drought in the U.S. Great Plains. Journal of Climate, 17(3), 485–503. https://doi.org/10.1175/1520-0442(2004)017%3C0485:COLDIT%3E2.0.CO;2

[wcc522-bib-0118] Schubert, S. D. , Suarez, M. J. , Pegion, P. J. , Koster, R. D. , & Bacmeister, J. T. (2004b). On the cause of the 1930s Dust Bowl. Science, 303(5665), 1855–1859. https://doi.org/10.1126/science.1095048 15031502 10.1126/science.1095048

[wcc522-bib-0119] Schurer, A. , Hegerl, G. , Mann, M. , Tett, S. , & Phipps, S. (2013). Separating forced from chaotic climate variability over the past millennium. Journal of Climate, 26, 6954–6973. https://doi.org/10.1175/JCLI-D-12-00826.1

[wcc522-bib-0120] Schurer, A. , Hegerl, G. , Ribes, A. , Polson, D. , Morice, C. , & Tett, S. (2018). *Estimating the transient climate response from observed warming. Journal of Climate* (under review).

[wcc522-bib-0121] Schurer, A. P. , Hegerl, G. C. , & Obrochta, S. P. (2015). Determining the likelihood of pauses and surges in global warming. Geophysical Research Letters, 42, 5974–5982. https://doi.org/10.1002/2015GL064458

[wcc522-bib-0122] Schurer, A. P. , Tett, S. F. B. , & Hegerl, G. C. (2014). Small influence of solar variability on climate over the past millennium. Nature Geoscience, 7, 104–108. https://doi.org/10.1038/ngeo2040

[wcc522-bib-0123] Seager, R. (2007). The turn of the century North American drought: Global context, dynamics, and past analogs. Journal of Climate, 20, 5527–5552. https://doi.org/10.1175/2007JCLI1529.1

[wcc522-bib-0124] Seager, R. , Kushnir, Y. , Herweijer, C. , Naik, N. , & Velez, J. (2005). Modeling of tropical forcing of persistent droughts and pluvials over Western North America: 1856–2000. Journal of Climate, 18, 4065–4088.

[wcc522-bib-0125] Seager, R. , Kushnir, Y. , Ting, M. , Cane, M. , Naik, N. , & Miller, J. (2008). Would advance knowledge of 1930s SSTs have allowed prediction of the Dust Bowl drought? Journal of Climate, 21(13), 3261–3281. https://doi.org/10.1175/2007JCLI2134.1

[wcc522-bib-0126] Shiogama, H. , Nagashima, T. , Yokohata, T. , Crooks, S. A. , & Nozawa, T. (2006). Influence of volcanic activity and changes in solar irradiance on surface air temperatures in the early twentieth century. Geophysical Research Letters, 33, L09702 https://doi.org/10.1029/2005GL025622

[wcc522-bib-0127] Sinha, A. , Kathayat, G. , Cheng, H. , Breitenbach, S. F. M. , Berkelhammer, M. , Mudelsee, M. , … Edwards, R. L. (2015). Trends and oscillations in the Indian summer monsoon rainfall over the last two millennia. Nature Communications, 6, 6309 https://doi.org/10.1038/ncomms7309 10.1038/ncomms7309 25686877

[wcc522-bib-0128] Sontakke, N. A. , Singh, N. , & Singh, H. N. (2008). Instrumental period rainfall series of the Indian region (AD 1813–2005): Revised reconstruction, update and analysis. The Holocene, 18, 1055–1066.

[wcc522-bib-0129] Srivastava, A. , & DelSole, T. (2017). Decadal predictability without ocean dynamics. Proceedings of the National Academy of Sciences, 114(9), 2177–2182. https://doi.org/10.1073/pnas.1614085114 10.1073/pnas.1614085114 PMC5338527 28193900

[wcc522-bib-0130] Steig, E. J. , Ding, Q. , White, J. W. C. , Küttel, M. , Rupper, S. B. , Neumann, T. A. , … Korotkikh, E. (2013). Recent climate and ice‐sheet changes in West Antarctica compared with the past 2,000 years. Nature Geoscience, 6, 372–375. https://doi.org/10.1038/ngeo1778

[wcc522-bib-0131] Stevens, B. (2015). Rethinking the lower bound on aerosol radiative forcing. Journal of Climate, 28, 4794–4819. https://doi.org/10.1175/JCLI-D-14-00656.1

[wcc522-bib-0132] Stott, P. A. , Christidis, N. , Otto, F. E. L. , Sun, Y. , Vanderlinden, J. , Van Oldenborgh, G. J. , … Zwiers, F. W. (2016). Attribution of extreme weather and climate‐related events. WIREs Climate Change, 7, 23–41. https://doi.org/10.1002/wcc.380 26877771 10.1002/wcc.380 PMC4739554

[wcc522-bib-0133] Sun, C. , Li, J. , & Jin, F. F. (2015). A delayed oscillator model for the quasi‐periodic multidecadal variability of the NAO. Climate Dynamics, 45, 2083–2099. https://doi.org/10.1007/s00382-014-2459-z

[wcc522-bib-0134] Sutton, R. T. , & Hodson, D. L. R. (2005). Atlantic Ocean forcing of North American and European summer climate. Science, 309, 115–119. https://doi.org/10.1126/science.1109496 15994552 10.1126/science.1109496

[wcc522-bib-0135] Thompson, D. M. , Cole, J. E. , Shen, G. T. , Tudhope, A. W. , & Meehl, G. A. (2015). Early twentieth‐century warming linked to tropical Pacific wind strength. Nature Geoscience, 8, 117–121. https://doi.org/10.1038/NGEO2321

[wcc522-bib-0136] Thompson, D. W. J. , Kennedy, J. J. , Wallace, J. M. , & Jones, P. D. (2008). A large discontinuity in the mid‐twentieth century in observed global‐mean surface temperature. Nature, 453, 646–649. https://doi.org/10.1038/nature06982 18509442 10.1038/nature06982

[wcc522-bib-0137] Ting, M. , Kushnir, Y. , & Li, C. (2014). North Atlantic multidecadal SST Oscillation: External forcing versus internal variability. Journal of Marine Systems, 133, 27–38. https://doi.org/10.1016/j.jmarsys.2013.07.006

[wcc522-bib-0138] Tokinaga, H. , Xie, S.‐P. , Deser, C. , Kosaka, Y. , & Okumura, Y. M. (2012). Slowdown of the Walker circulation driven by tropical Indo‐Pacific warming. Nature, 491(7424), 439–443. https://doi.org/10.1038/nature11576 23151588 10.1038/nature11576

[wcc522-bib-0139] Tokinaga, H. , Xie, S.‐P. , & Mukougawa, H. (2017). Early 20th‐century Arctic warming intensified by Pacific and Atlantic multidecadal variability. Proceedings of the National Academy of Sciences, 114(24), 6227–6232. https://doi.org/10.1073/pnas.1615880114 10.1073/pnas.1615880114 PMC5474770 28559341

[wcc522-bib-0140] Trenberth, K. E. (2015). Has there been a hiatus? Science, 349(6249), 691–692. https://doi.org/10.1126/science.aac9225 26273042 10.1126/science.aac9225

[wcc522-bib-0141] Trenberth, K. E. , & Shea, D. J. (2006). Atlantic hurricanes and natural variability in 2005. Geophysical Research Letters, 33, L12704 https://doi.org/10.1029/2006GL026894

[wcc522-bib-0142] Tung, K.‐K. , & Zhou, J. (2013). Using data to attribute episodes of warming and cooling in instrumental records. Proceedings of the National Academy of Sciences, 6, 2058–2063. https://doi.org/10.1073/pnas.1212471110 10.1073/pnas.1212471110 PMC3568361 23345448

[wcc522-bib-0143] Ummenhofer, C. C. , England, M. H. , Mclntosh, P. C. , Meyers, G. A. , Pook, M. J. , Risbey, J. S. , … Taschetto, A. S. (2009). What causes Southeast Australia's worst droughts? Geophysical Research Letters, 36, L04706 https://doi.org/10.1029/2008GL036801

[wcc522-bib-0144] Undorf, S. , Bollasina, M. A. , & Hegerl, G. C. (2018). Impacts of the 1900‐1974 increase in anthropogenic aerosol emissions from North America and Europe on northern hemisphere summer climate. Journal of Climate (under review).

[wcc522-bib-0145] Undorf, S. , Polson, D. , Bollasina, M. , Ming, Y. , Schurer, A. , & Hegerl, G. C. (2018). Detectable impact of local and remote anthropogenic aerosols on the 20th century changes of West African and South Asian monsoon precipitation. Journal of Geophysical Research (under review).

[wcc522-bib-0146] Verdon‐Kidd, D. C. , & Kiem, A. S. (2009). Nature and causes of protracted droughts in Southeast Australia: Comparison between the Federation, WWII, and Big Dry droughts. Geophysical Research Letters, 36, L22707 https://doi.org/10.1029/2009GL041067

[wcc522-bib-0147] Walsh, J. E. , Fetterer, F. , Stewart, J. S. , & Chapman, W. L. (2017). A database for depicting Arctic Sea ice variations back to 1850. Geographical Review, 107, 89–107. https://doi.org/10.1111/j.1931-0846.2016.12195.x

[wcc522-bib-0148] Wang, B. (2006). The Asian monsoon. Berlin and Heidelberg, Germany; New York, NY: Springer.

[wcc522-bib-0149] Wang, Y. , Lean, J. L. , & Sheeley, N. R., Jr. (2005). Modelling the Sun's magnetic field and irradiance since 1713. The Astrophysical Journal, 625, 522–538.

[wcc522-bib-0150] Wegmann, M. , Brönnimann, S. , & Compo, G. P. (2017). Tropospheric circulation during the early twentieth century Arctic warming. Climate Dynamics, 48(7), 2405–2418. https://doi.org/10.1007/s00382-016-3212-6

[wcc522-bib-0151] Wood, K. R. , & Overland, J. E. (2010). Early 20th century Arctic warming in retrospect. International Journal of Climatology, 30, 1269–1279. https://doi.org/10.1002/joc.1973

[wcc522-bib-0152] Woodhouse, C. A. , & Overpeck, J. T. (1998). 2000 Years of drought variability in the Central United States. Bulletin of the American Meteorological Society, 1998(79), 2693–2714.

[wcc522-bib-0153] Worster, D. (1979). Dust Bowl: The Southern Plains in the 1930s. New York, NY: Oxford University Press.

[wcc522-bib-0154] Yamanouchi, T. (2011). Early 20th century warming in the Arctic: A review. Polar Science, 5, 53–71.

[wcc522-bib-0155] Yoshimori, M. , Stocker, T. , Raible, C. , & Renold, M. (2005). Externally forced and internal variability in ensemble climate simulations of the maunder minimum. Journal of Climate, 18, 4253–4270. https://doi.org/10.1175/JCLI3537.1

[wcc522-bib-0156] Zhang, R. , Delworth, T. , Sutton, R. , Hodson, D. , Dixon, K. , Held, I. , … Vecchi, G. (2013). Have aerosols caused the observed Atlantic multidecadal variability? Journal of Atmospheric Sciences, 70, 1135–1144. https://doi.org/10.1175/JAS-D-12-0331.1

[wcc522-bib-0157] Zhang, Y. , Wallace, J. , & Battisti, D. (1997). ENSO‐like Interdecadal variability: 1900–93. Journal of Climate, 10, 1004–1020. https://doi.org/10.1175/1520-0442(1997)010%3C1004:ELIV%3E2.0.CO;2

[wcc522-bib-0158] Zhou, T. , Brönnimann, S. , Griesser, T. , Fischer, A. M. , & Zou, L. (2010). A reconstructed dynamic Indian monsoon index extended back to 1880. Climate Dynamics, 34, 573–585. https://doi.org/10.1007/s00382-009-0552-5

