# Supplementary material for: The early 20th century warming: Anomalies, causes, and consequences
Source: Wiley Interdiscip Rev Clim Change. 2018 Apr 25;9(4):e522. doi: 10.1002/wcc.522 (PMC6033150; doi:10.1002/wcc.522)
Supplement: Supplementary file 1 — Appendix S1. [file WCC-9-na-s001.docx]

**Supplementary Information**

This supplement contains additional information on figures made that go beyond the information in the body of text, and some additional figures for further information or sensitivity analysis. For references see body of paper.

**Background information for figure1 and subsequent figures:**

These figures follow Schurer et al 2018, and are based on variants of multi-model analysis and detection and attribution methods used e.g. in the IPCC report (Bindoff et al., 2013). The observations used are HadSST3.1.1.0 for oceans, CRUTEM4.5.0.0 for land and HadCRUT4.5.0.0 for the combined ocean and land composite.

Global annual temperatures are caculated using the same method as done by the Met Office, namely (NH+SH)/2 for HadSST and HadCRUT and (NH*2 + SH)/3 for CRUTEM. All anomlies are plotted with respect to 1870-2013.

The model temperatures are calculated using the sea surface temperature over ocean and surface air temperature over land masked to the observations. The blend of SSTs and SATs for the combined land and ocean composite (to compare to HadCRUT4) is calculated following Cowtan et al 2015.

For all plots the model temperatures are taken as the annual multi-model-mean used in Schurer et al 2018, which only includes models with the indirect aerosol effect and cover the period 1863-2012. This multi-model mean is regressed onto the median observered temperature using a total least squares regression, and the multi-model mean is plotted multiplied by this scaling factor.

The calculated scaling factors, which indicates by what factor raw multi-model fingerprints have to be multiplied to replicate the observed response to all forcings combined, are close to unity (**1.06** for CRUTEM, **0.96** for HadSST and **1.01** for HadCRUT4.5.0), indicating that the multimodel mean all forced fingerprints is well supported by observations and does not need adjustment.

For the land and ocean (panel c) the reconstruction from Crowley et al 2014 is also included. This has data up until 1984. Anomalies for this are calculated to be equal to the anomlies in HadCRUT4 over the shared time period (1850-1984).

The residual is calculated as the difference between the observations and the scaled multi-model mean. This residual is divided by a factor (1.03) which accounts for the small amound of internal variability that remains after averaging in the model simulations.

Plus or minus two times the standard deviation from all the preindustrial control (piControl) simulations used in Schurer et al 2018 is shown in purple in figure d. The picontrol simulations are calculated as a masked blend of SATs and SSTs to match the observations.

**Figure 2**

Panels (a) and (b) show decadal trends in HadCRUT4.5.0.0. The cold and warm seasons are defined based on the boreal hemisphere as October-March, and April-September. Grid cells are masked (in grey) where coverage in either the first or second half of the period drops below 30%.

Panels (c) and (d) are spatial plots of the anomalies in residual temperature between observered and modelled temperature.

For the residual the modelled contribution is calculated using the surface air temperture field as the mean over ensemble means, where each ensemble mean is first masked, anomalies and then the spatial field is scaled to obseved temperature (HadCRUT4.5.0.0) by regressing the global mean temperature against the global mean observed temperature using a total least squares regression..

The models used are listed in table S1, with details of the number of ensemble members each mofdel has the the regression factor used to scale the model to observations. Anomalies are taken over the period 1870-2013.

| **Model** | **Number of Ensemble members** | **Calculated scaling factor** | | |
| --- | --- | --- | --- | --- |
|  |  | **CRUTEM** | **HadSST** | **HadCRUT4** |
| ACCESS1-0 | 1 | 1.0 | 1.2 | 1.0 |
| ACCESS1-3 | 1 | 1.6 | 1.5 | 1.4 |
| BNU-ESM | 1 | 0.6 | 0.6 | 0.6 |
| CCSM4 | 6 | 0.8 | 0.6 | 0.7 |
| CESM1-BGC | 1 | 0.9 | 0.7 | 0.7 |
| CESM1-CAM5 | 3 | 1.3 | 1.1 | 1.1 |
| CMCC-CMS | 1 | 1.3 | 1.2 | 1.2 |
| CMCC-CM | 1 | 1.3 | 1.2 | 1.2 |
| CNRM-CM5 | 10 | 1.1 | 0.9 | 1.0 |
| CSIRO-Mk3-6-0 | 10 | 1.4 | 1.1 | 1.2 |
| CanESM2 | 5 | 0.9 | 0.7 | 0.7 |
| EC-EARTH | 4 | 0.8 | 0.7 | 0.8 |
| FIO-ESM | 3 | 1.0 | 0.8 | 0.8 |
| GFDL-CM2p1 | 10 | 0.8 | 0.7 | 0.7 |
| GFDL-CM3 | 1 | 1.2 | 1.0 | 1.0 |
| GFDL-ESM2G | 1 | 1.0 | 0.8 | 0.9 |
| GFDL-ESM2M | 1 | 1.0 | 0.9 | 0.9 |
| GISS-E2-H-CC | 1 | 0.9 | 0.7 | 0.8 |
| GISS-E2-H (p1) | 5 | 1.1 | 0.8 | 0.9 |
| GISS-E2-H (p2) | 4 | 1.3 | 1.0 | 1.0 |
| GISS-E2-H (p3) | 5 | 0.9 | 0.75 | 0.8 |
| GISS-E2-R-CC | 1 | 1.3 | 1.0 | 1.1 |
| GISS-E2-R (p1) | 4 | 1.2 | 0.9 | 1.0 |
| GISS-E2-R (p2) | 4 | 1.5 | 1.2 | 1.3 |
| GISS-E2-R (p3) | 4 | 1.1 | 0.9 | 0.9 |
| HadCM3 | 10 | 0.0 | 0.4 | 0.1 |
| HadGEM2-AO | 1 | 1.0 | 0.9 | 0.9 |
| HadGEM2-CC | 1 | 0.0 | 0.3 | 0.0 |
| HadGEM2-ES | 4 | 0.0 | 0.3 | 0.0 |
| IPSL-CM5A-LR | 4 | 0.7 | 0.6 | 0.6 |
| IPSL-CM5A-MR | 1 | 0.9 | 0.7 | 0.7 |
| IPSL-CM5B-LR | 1 | 0.9 | 0.7 | 0.8 |
| MIROC-ESM-CHEM | 1 | 1.4 | 1.0 | 1.0 |
| MIROC-ESM | 1 | 1.3 | 0.9 | 1.0 |
| MIROC5 | 5 | 1.3 | 1.0 | 1.1 |
| MPI-ESM-LR | 3 | 0.8 | 0.7 | 0.7 |
| MPI-ESM-MR | 3 | 0.8 | 0.7 | 0.7 |
| MRI-CGCM3 | 3 | 1.5 | 1.5 | 1.5 |
| NorESM1-ME | 1 | 1.2 | 1.1 | 1.1 |
| NorESM1-M | 3 | 1.3 | 1.1 | 1.1 |
| bcc-csm1-1-m | 3 | 0.8 | 0.7 | 0.7 |
| bcc-csm1-1 | 3 | 0.8 | 0.7 | 0.7 |
| inmcm4 | 1 | 1.6 | 1.2 | 1.3 |

**Table S1: List of models used to make figures 2, 6, 10** with the number of ensemble members used in the scaling factor used to scale the ensemble mean to the observations.

**Figure 5**

This figure follows the analysis in Schurer et al 2018.

The analysis calculates likelihoods for the scaling factors $\beta$:

$$Y\left( t \right)={X_{historical}}^{*}\beta_{historical}+{{X_{historicalGHG}}^{*}\beta}_{historicalGHG}+{{X_{historicalNAT}}^{*}\beta}_{historicalNAT}+\varepsilon_{0}$$

Where Y is the observated temperature (HadCRUT4.5.0.0), $X$ are the CMIP5 multi-model mean temperature simulations, for the all forced, greenhouse gas only and natural forced only experiment and $\varepsilon_{0}$ is internal variability. For each combination of scaling factors, $\beta,$ the 1901-1950 warming trend is calcualted and a likelihood is assigned to this trend according to the likelihood of the scaling factors given the observed temperatures. These likelihoods are calculated using fingerprints of global mean and hemispheric difference and use the variance adjusted analysis described in Schurer et al 2017. Results are presented for both the case using informed prior information and an unifomed prior. For control simulations supporting the green histogram, the same control simulations have been used as for Figure 6.

**Figure 6**

The annual spatial residual is calculated using the same analysis technnique as in figure 2 i.e. the mean of scaled ensemble means which have first been masked and scaled to observations of land-only (CRUTEM4.5.0.0), ocean-only (HadSST3.1.1.0) and combined land and ocean (HadCRUT4.5.0.0). Details of model used given in table S1. The zonal mean temperature is calculatd from these residuals.

In each zonal band this residual is divided by internal variability. This is calculated from the control simulations listed in table S2. For each year in the residual timeseries a mask is derived to account for monthly observational coverage. Then each of the control simulations is masked to the observed coverage and annual zonal means are calculated, from which the standard deviation of annual zonal means is calculated for each year of each control simulations and an average standard deviation is calculated accros all model control simulations.

All data is further masked so that each zonal band has at least 10% coverage in each of the seasons which make up the annual means, with Figure S6 illustrating that sharpening the data criterion to 30% coverage does not affect key conclusions.

| **CMIP5 piControl simulations** |
| --- |
| ACCESS1-3_piControl_r1i1p1 |
| BNU-ESM_piControl_r1i1p1 |
| CCSM4_piControl_r1i1p1 |
| CCSM4_piControl_r2i1p1 |
| CCSM4_piControl_r3i1p1 |
| CESM1-BGC_piControl_r1i1p1 |
| CESM1-CAM5_piControl_r1i1p1 |
| CESM1-FASTCHEM_piControl_r1i1p1 |
| CESM1-WACCM_piControl_r1i1p1 |
| CMCC-CESM_piControl_r1i1p1 |
| CMCC-CMS_piControl_r1i1p1 |
| CMCC-CM_piControl_r1i1p1 |
| CNRM-CM5-2_piControl_r1i1p1 |
| CNRM-CM5-2_piControl_r1i1p2 |
| CNRM-CM5-2_piControl_r1i1p3 |
| CNRM-CM5-2_piControl_r1i1p4 |
| CNRM-CM5_piControl_r1i1p1 |
| ACCESS1-0_piControl_r1i1p1 |
| CSIRO-Mk3-6-0_piControl_r1i1p1 |
| CSIRO-Mk3L-1-2_piControl_r1i1p1 |
| CanESM2_piControl_r1i1p1 |
| FGOALS-g2_piControl |
| FGOALS-s2_piControl |
| FIO-ESM_piControl_r1i1p1 |
| GFDL-CM3_piControl_r1i1p1 |
| GFDL-ESM2G_piControl_r1i1p1 |
| GFDL-ESM2M_piControl_r1i1p1 |
| GISS-E2-H-CC_piControl_r1i1p1 |
| GISS-E2-H_piControl_r1i1p1 |
| GISS-E2-H_piControl_r1i1p2 |
| GISS-E2-H_piControl_r1i1p3 |
| GISS-E2-R-CC_piControl_r1i1p1 |
| GISS-E2-R_piControl_r1i1p141 |
| GISS-E2-R_piControl_r1i1p142 |
| GISS-E2-R_piControl_r1i1p1 |
| GISS-E2-R_piControl_r1i1p2 |
| GISS-E2-R_piControl_r1i1p3 |
| HadGEM2-AO_piControl_r1i1p1 |
| HadGEM2-CC_piControl_r1i1p1 |
| HadGEM2-ES_piControl_r1i1p1 |
| IPSL-CM5A-LR_piControl_r1i1p1 |
| IPSL-CM5A-MR_piControl_r1i1p1 |
| IPSL-CM5B-LR_piControl_r1i1p1 |
| MIROC-ESM-CHEM_piControl_r1i1p1 |
| MIROC-ESM_piControl_r1i1p1 |
| MIROC4h_piControl_r1i1p1 |
| MIROC5_piControl_r1i1p1 |
| MPI-ESM-LR_piControl_r1i1p1 |
| MPI-ESM-MR_piControl_r1i1p1 |
| MPI-ESM-P_piControl_r1i1p1 |
| MRI-CGCM3_piControl_r1i1p1 |
| NorESM1-ME_piControl_r1i1p1 |
| NorESM1-M_piControl_r1i1p1 |
| bcc-csm1-1-m_piControl_r1i1p1 |
| bcc-csm1-1_piControl_r1i1p1 |
| inmcm4_piControl_r1i1p1 |

**Table S2 CMIP5 piControl simulations used to calculate zonal internal variability for figure 6**

**Further figures and sensitivity tests:**


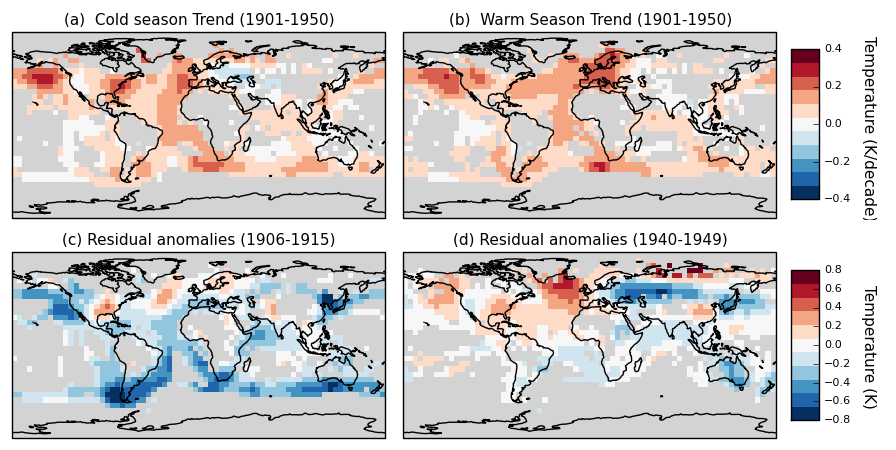
Figure S1: As figure 2, but showing the period 1901-1950 on top which is used in the analysis in figure 5.

Figure S2: As figure 2 in the body of the paper, and on same colourscale, but showing multimodel mean CMIP5 forced decadal temperature trends for boreal cold (Nov-March) and warm season (April-October), for only anthropogenic forcings (a and b) and only natural forcings (c and d).


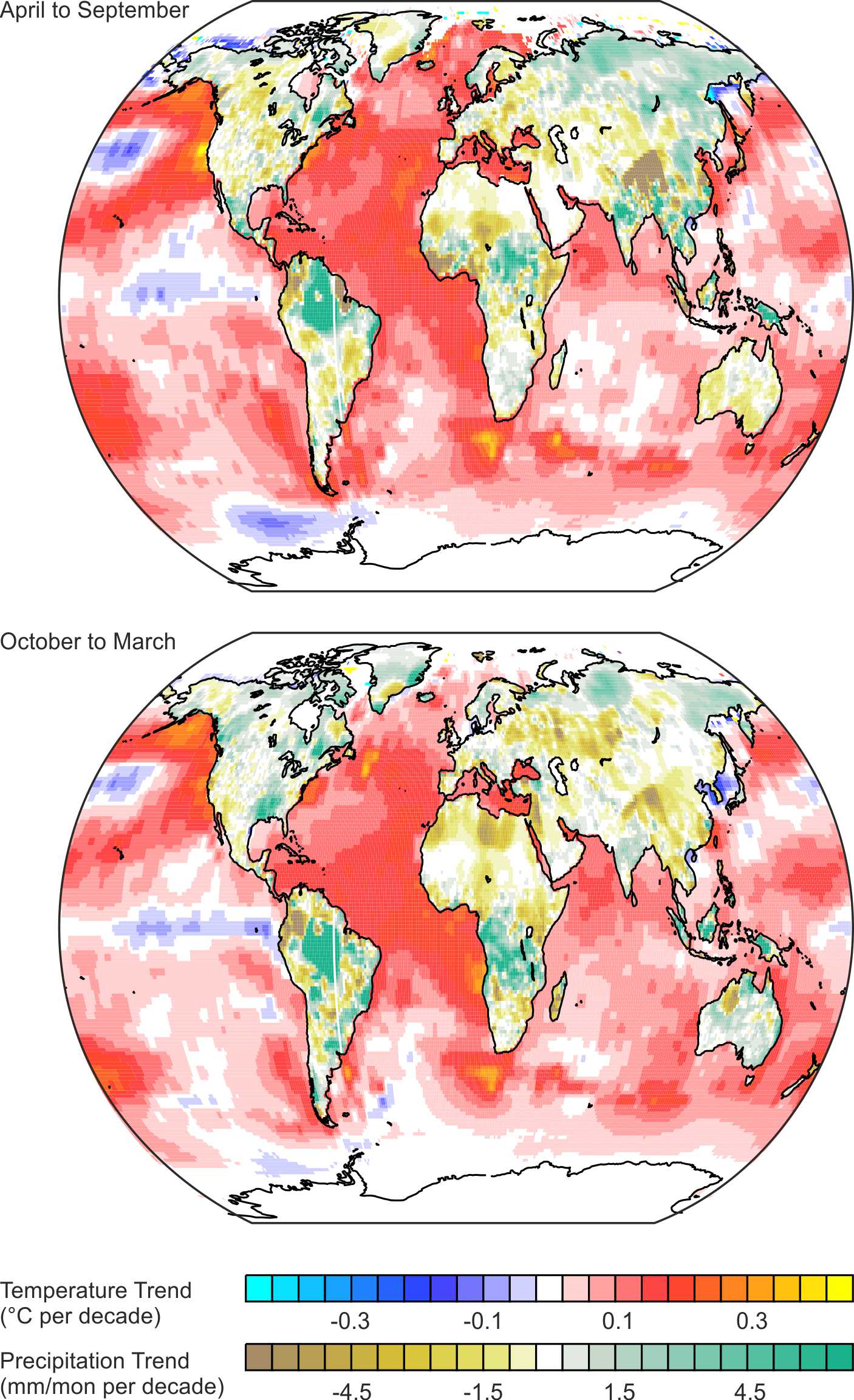


Figure S3: Trends in sea-surface temperature and land precipitation from the Global Precipitation Climatology Centre (GPCC) observations, 1901-1945. The data are based on station data but infilled where these show gaps, hence there are substantial uncertainties due to infilled data. Trends were calculated with least squares regression from seasonal averages of sea-surface temperature (HadISST1) and precipitation (GPCC reanalysis).


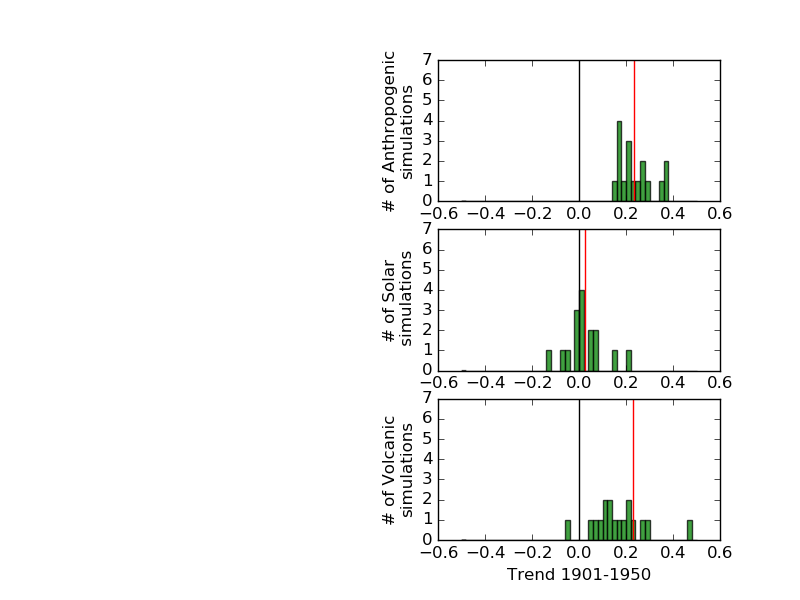


Figure S4: Global mean trends for 1901-1950 from individual simulations with anthropogenic, solar and volcanic forcings separately (as available in the CMIP5 archive). The average of the ensemble mean of each of the models who ran such individual simulations is shown by the red line, indicating an average anthropogenic warming of 0.24K over the 1901-1950 period, solar 0.025K, volcanic 0.23K, with largest uncertainty in the contribution by natural forcings.


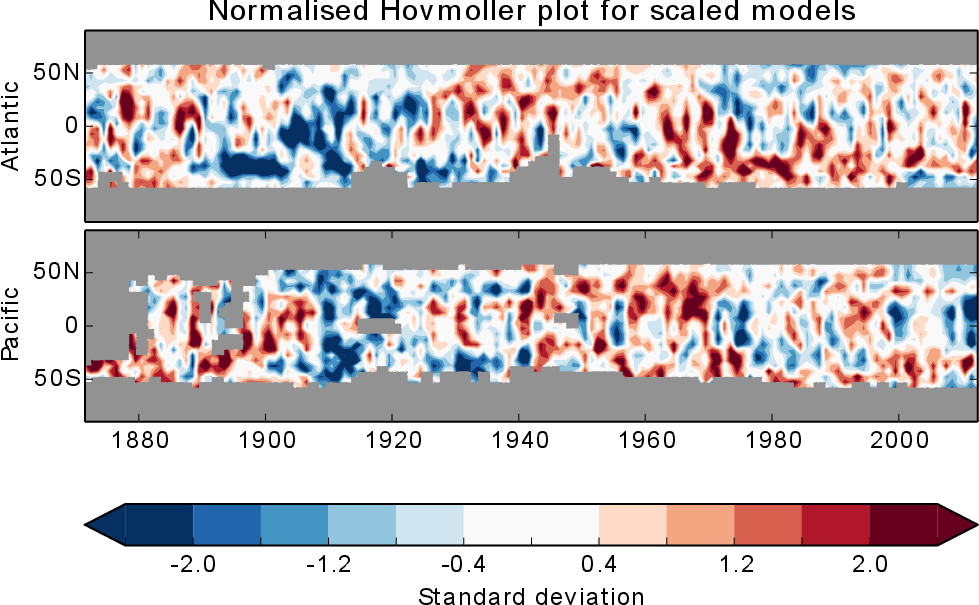


Figure S5: Hovmoeller diagram of standardized zonal mean sea surface temperature anomalies separately for Atlantic (top – defined as longitude 110W-10W and latitude 60S-60N) and Pacific basin (bottom – defined as longitude 140E-260E and latitude 60S-60N)


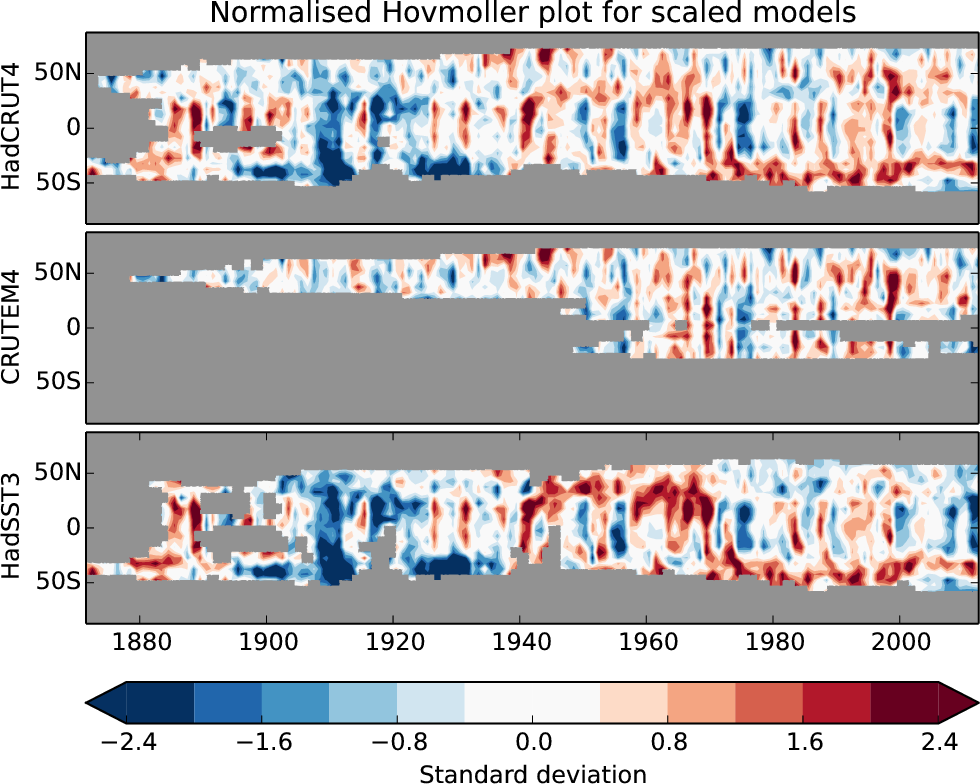


Figure S6: Hovmoeller diagram of standardized zonal mean temperature anomalies – Same as figure 6 except that model data is masked where data coverage for a particular zonal band drops below 30% in any season.


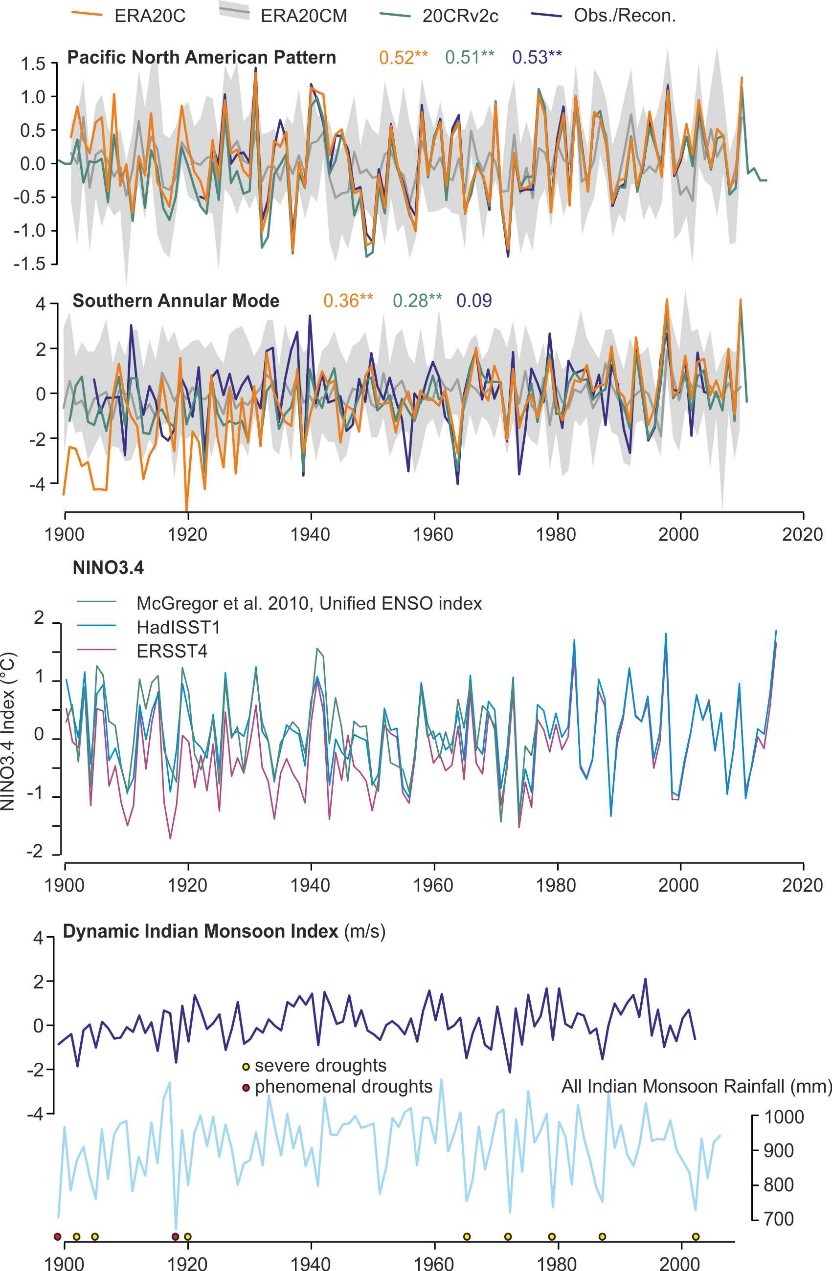


Figure S7: Annual time series of the Pacific North American index (Dec.-Feb.), the Southern Annular Mode (Jun.-Aug.), NINO3.4 (Sep.-Jan.), the Dynamic Indian Monsoon index (Zhou et al. 2010), and Indian Summer monsoon rainfall (Sontakke et al. 2008) indices (both Jun.-Aug.), expressed as anomalies from 1961-1990. Shown are series from two reanalyses (20CRv2c, ERA20C), the atmospheric model simulations ERA20CM (10 members; ensemble mean and spread are indicated) and series based on observations or reconstructions. Coloured numbers indicate correlations between the correspondingly coloured reanalyses or observations with the ERA20CM ensemble mean (* and ** denote 95% or 99% significance). Severe and phenomenal droughts over India (according to Wang, 2006) are added as circles.


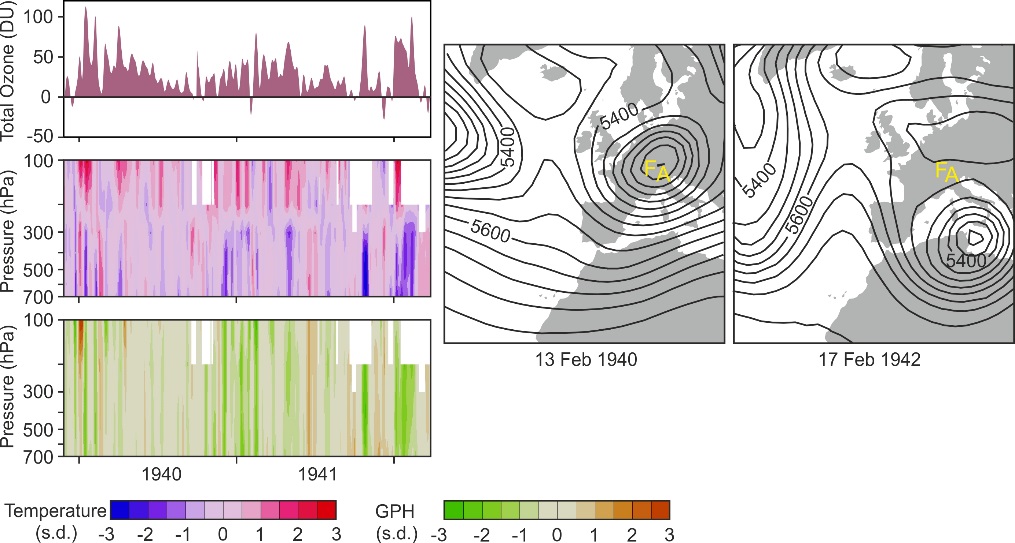


Fig. S8. Cold winters during World War II. Anomalously cold winters occur 1939/40, 40/41, 41/42 and 46/47, linked to anomalous circulation and strong blocking events. From Brönnimann S. & Compo G. P. (2012) Ozone highs and associated flow features in the first half of the twentieth century in different data sets. Reprinted with permission from *Meteorologische Zeitschrift*, 21, 49–59, doi: 10.1127/0941-2948/2012/0284 (available from https://www.schweizerbart.de/journals/metz).


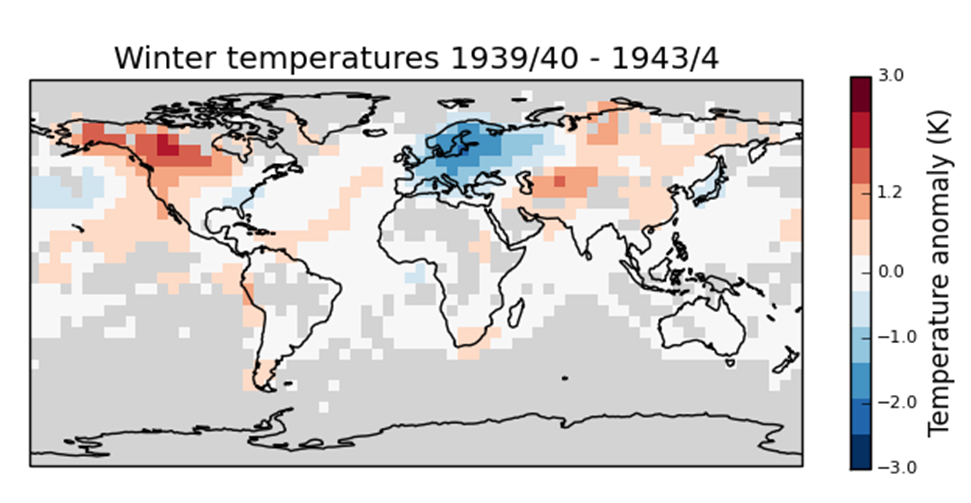


Figure S9: Anomalously cold winters over Europe averaged over the early 1940s (DJF average from 1939-40 to 1943/44) influenced by the blocking events shown in Figure S3. Anomalies are calculated from winters in the period 1920-1964. Regions are masked where coverage across the whole period drops below 30%.
